# Prevalence, risk factors and burden of diabetic retinopathy in China: a systematic review and meta-analysis

**DOI:** 10.7189/jogh.08.010803

**Published:** 2018-06

**Authors:** Peige Song, Jinyue Yu, Kit Yee Chan, Evropi Theodoratou, Igor Rudan

**Affiliations:** 1 Centre for Global Health Research, Usher Institute of Population Health Sciences and Informatics, University of Edinburgh, Edinburgh, Scotland, United Kingdom; 2 UCL Great Ormond Street Institute of Child Health, University College London, London, United Kingdom

## Abstract

**Background:**

Diabetic retinopathy (DR), the primary retinal vascular complication of diabetes mellitus (DM), is a leading cause of vision impairment and blindness in working-age population globally. Despite mounting concerns about the emergence of DM as a major public health problem in the largest developing country, China, much remains to be understood about the epidemiology of DR. We aimed to investigate the prevalence of and risk factors for DR, and estimate the burden of DR in China in 2010.

**Methods:**

China National Knowledge Infrastructure (CNKI), Wanfang, Chinese Biomedicine Literature Database (CBM-SinoMed), PubMed, Embase and Medline were searched for studies that reported the prevalence of and risk factors for DR in Chinese population between 1990 and 2017. A random-effects meta-analysis model was adopted to pool the overall prevalence of DR. Variations in the prevalence of DR in different age groups, DM duration groups and settings were assessed by subgroup meta-analysis and meta-regression. Odds ratios (ORs) of major risk factors were pooled using random-effects meta-analysis. The number of people with DR in 2010 was estimated by multiplying the age-specific prevalence of DR in people with DM with the corresponding number of people with DM in China. Finally, the national number of people with DR was distributed into six geographic regions using a risk factor-based model.

**Results:**

A total of 31 studies provided information on the prevalence of DR and 21 explored potential risk factors for DR. The pooled prevalence of any DR, nonproliferative DR (NPDR) and proliferative DR (PDR) was 1.14% (95% CI = 0.80-1.52), 0.90% (95% CI = 0.56-1.31) and 0.07% (95% CI = 0.02-0.14) in general population; In people with DM, the pooled prevalence rates were 18.45% (95% CI = 14.77-22.43), 15.06% (95% CI = 11.59-18.88) and 0.99% (95% CI = 0.40-1.80) for any DR, NPDR and PDR, respectively. The prevalence of any DR in DM patients peaked between 60 and 69 years of age, and increased steeply with the duration of DM. DM patients residing in rural China were at a higher risk to have DR than those in urban areas. In addition, insulin treatment, elevated FBG level and higher HbA1c concentration were confirmed to be associated with a higher prevalence of DR in people with DM, with meta-ORs of 1.99 (95% CI = 1.34-2.95), 1.33 (95% CI = 1.12-1.59) and 1.15 (95% CI = 1.09-1.20) respectively. In 2010, a total of 13.16 million (95% CI = 8.95-18.00) Chinese aged 45 years and above were living with DR, among whom the most were in South Central China and the least were in Northwest China.

**Conclusions:**

DR has become a serious public health problem in China. Optimal screening of and interventions on DR should be implemented. Improved epidemiological studies on DR are still required.

Diabetic retinopathy (DR), the primary retinal vascular complication of diabetes mellitus (DM), is a leading cause of vision impairment and blindness in the working-age population [1-4]. In the early course of the disease, DR is generally asymptomatic. If left untreated, DR can seriously impair vision, and eventually progress to blindness [1,3]. Apart from its devastating visual effects that might lead to reduced mobility, depression and lower quality of life, DR is also associated with a higher risk of systemic vascular complications, imposing a noteworthy burden on individuals, households, communities and societies [5-7]. DR is a progressive disease that can be broadly divided into two stages according to its severity: nonproliferative and proliferative. Nonproliferative DR (NPDR) is characterized by microaneurysms, cotton-wool spots, intraretinal microvascular abnormalities, hard exudates and venous beading, whereas proliferative DR (PDR) is hallmarked by neovascularization of the optic disc or elsewhere, pre-retinal and vitreous haemorrhage [1,8]. Taken individually, PDR is less common but more sight-threatening than NPDR [1-3,8,9].

Although available diagnostic and therapeutic advancements, such as optimum management of DM and early detection of DR, can substantially reduce the risk of visual deterioration, DR remains an important cause of visual impairment and blindness globally [10-16]. In 2010, 3.7 million people were visually impaired and 0.8 million were blind because of DR, accounting for 1.9% of all visually impaired cases and 2.6% of all blind cases worldwide [13]. With DM having reached epidemic proportions worldwide, estimating the prevalence of DR in both the general population and those with DM is imperative for driving better health policy making and improved programming [13,15]. By pooling data from 35 population-based studies across the world, the Global DR Study estimated that the prevalence of any DR, PDR and vision-threatening DR (severe retinopathy and macular oedema) were 34.6%, 7.0% and 10.2% respectively among individuals with DM, translating to approximately 93 million people with any DR, 17 million with PDR and 28 million with vision-threatening DR worldwide in 2010 [2]. Unless substantial improvements occur in the prevention and treatment of DR, the prevalence and burden of DR will continue to escalate as the global population ages and the epidemic of DM expands [17-19]. In addition, evaluation of risk factors for DR is of special importance in optimal clinical management. Similar to other common complications of DM, DR is a sentinel indicator of the progression of DM, thus its prevalence, not surprisingly, associated with the duration and severity of DM [2,4,20]. In the Global DR Study, longer DM duration has been recognised as a key risk factor for DR in people with DM, as well as higher levels of haemoglobin A1c (HbA1c) and blood pressure. Moreover, individuals with type 1 DM (T1DM) are more likely to develop DR than those with type 2 DM (T2DM) [2,4].

Despite mounting concerns about the emergence of DM as a major public health problem in the largest developing country, China, epidemiological data on DR in Chinese population are still rather scarce or inconsistent [9,21-23]. Thus far, there is still no national population-based data on the prevalence and burden of DR in China, and the existing surveys on DR are restricted to local characteristics, study methodologies, ascertainment and classification of DR, limiting direct comparisons between individual studies [22]. A systematic review and meta-analysis by Liu and colleagues, dating back to 2012, has provided the first overview of the DR prevalence in China. Based on 19 individual studies, their meta-analysis suggested that the pooled prevalence rates of any DR, NPDR and PDR in general Chinese population were 1.3%, 1.1%, and 0.1% and those in people with DM were 23.0%, 19.1%, and 2.8% respectively [22]. Thereafter, a growing body of epidemiological data on DR has become available in China, yet virtually none of them has been systematically appraised, underscoring the need for an updated analysis [24,25]. Moreover, the effects of major risk factors for DR are still discrepant and inconclusive among the Chinese population, which need to be systematically evaluated in an evidence-based fashion.

To fill the gaps outlined above, we conducted a comprehensive systematic review, in both Chinese and English databases, to retrieve studies that reported the epidemiology of DR in China from 1990 onwards. Based on the existing evidence, we aimed to: (1) pool the overall prevalence of DR in both general Chinese population and people with DM; (2) estimate the effects of demographic and geographic variables on the prevalence of DR in people with DM; (3) assess the major risk factors for DR in people with DM; and (4) quantify the national and subnational burden of DR in 2010.

## METHODS

### Systematic review

This systematic review and meta-analysis adheres to the Preferred Reporting Items for Systematic Reviews and Meta-Analyses (PRISMA) guidelines and the Guidelines for Accurate and Transparent Health Estimates Reporting (GATHER) statement [26,27].

#### Search strategy

Three Chinese and three English electronic bibliographic databases, namely China National Knowledge Infrastructure (CNKI), Wanfang, Chinese Biomedicine Literature Database (CBM-SinoMed), PubMed, Embase, and Medline, were searched to locate all relevant publications that reported the epidemiology of DR in China. Our comprehensive search strategies combined terms of diabetic retinopathy, epidemiology (incidence, prevalence, morbidity, mortality, epidemiology) and China (China, Chinese, Hong Kong, Macau, Taiwan) using both controlled vocabularies (eg, Medical Subject Heading terms) and free text words. Search queries were optimised to fit the specific features of each database, and the full search strategies are detailed in Table S1 in **Online Supplementary Document(Online Supplementary Document)**. To supplement the electronic database search, reference lists of eligible publications and related reviews were also scanned to identify other potentially pertinent studies. The literature search was limited to studies published between January 1990 and December 2017. No language restrictions were imposed on searches or search results.

#### Inclusion and exclusion criteria

To be included in the systematic review and meta-analysis, studies had to be population-based and reported the prevalence of DR or risk factors for DR. Depending on how the study population was sought, the identified population-based studies can be classified into three categories: community-based, primary health care management (PHCM)-based and registry-based. Community-based studies derived their study sample from the general population (eg, cluster sampling of households), whereas PHCM-based and registry-based studies derived their study sample from all the primary care settings or primary care systems in a defined geographical area. Thus, both PHCM-based and registry-based studies attempted to capture all, or at least a random sample, of people with DM in a defined geographical area. For the purpose of pooling prevalence rates of DR, the included studies must be community-based, and of particular note, include both newly detected and physician-diagnosed DM cases simultaneously (to avoid overestimation); To assess the risk factors for DR in people with DM, the included studies could be community-based, PHCM-based or registry-based, where DM cases could be either newly detected or physician-diagnosed, or both. To avoid suspected bias inherent to univariate analysis, the estimation of odds ratios (ORs) in studies that reported the risk factors for DR must be based on a multivariate study design.

Studies that were conducted in the T1DM group were excluded, whereas those focused on people with T2DM were retained. Studies that contained both T1DM and T2DM cases were not excluded if the proportion of people with T1DM was small (<10%). For studies where the type of DM was not specified but all other criteria were fulfilled, it was assumed that those studies contained both T2DM cases and a small proportion of T1DM cases. Otherwise, the type of DM could be speculated by the age at diagnosis of DM (if available), where people diagnosed before 30 years were deemed as with T1DM and those after 30 years were T2DM [2,28]. To be eligible for inclusion, studies must have undertaken fundus photography (FP) to ascertain DR and provided numerical estimates of DR prevalence. Reviews, commentaries and studies where the prevalence rates were calculated based on the number of eyes with DR rather than the number of affected individuals were excluded.

#### Study selection and data extraction

After deleting duplicate records within and between different bibliographic databases, the remaining titles and abstracts were independently reviewed by two authors (PS and JY) to identify potentially eligible articles that required a full appraisal. In cases of multiple publications from the same study or overlapping data, preference was given to the most recent one or the one with the most inclusive information. Consensus was achieved for any discrepancies in study eligibility through discussion. With a predefined data-collection form, the following information was extracted from the included studies, where possible:

1. Characteristics of the study: author(s), publication year, study year, study type (community-based, PHCM-based or registry-based), sampling method, study design (cross-sectional or cohort), study setting (urban, rural or mixed) and location, geographic region, DR assessment method and grading system;
2. Characteristics of the sample (general population and people with DM): number of the sample, age (age range, mean or median age), gender (male, female or mixed), DM definition, DM classification (T1DM or T2DM, newly detected or physician-diagnosed), and duration of DM;
3. Prevalence data: the number of people with DR and the number of participants who had been tested for DR, by age, DM duration, gender, setting and DR subtype;
4. Risk factor data: definition of risk factor, OR and corresponding confidence intervals (CIs).

According to the definitions from National Bureau of Statistics of China, the geographic regions where the studies were carried out were classified into six categories: East China, North China, Northeast China, Northwest China, South Central China, and Southwest China (see **Table 1**) [29,30]. Missing values for the median year of study were imputed by subtracting three years (the average time-lag from investigation to publication in studies with available data) from their publication years, and this was done for six individual studies. In this study, we further classified DR as NPDR and PDR. Therefore, relevant data were extracted from the included studies for different subtypes of DR respectively, wherever available.

**Table 1:** The six geographic regions in China

Region	Included provinces
North China	Beijing Municipality, Hebei province, Inner Mongolia Autonomous Region, Shanxi province, Tianjin Municipality
Northeast China	Heilongjiang province, Jilin province, Liaoning province
East China	Anhui province, Fujian province, Jiangsu province, Jiangxi province, Shandong province, Shanghai Municipality, Zhejiang province
South Central China	Guangdong province, Guangxi Zhuang Autonomous Region, Hainan province, Henan province, Hubei province, Hunan province
Southwest China	Chongqing Municipality, Guizhou province, Sichuan province, Tibet Autonomous Region, Yunnan province
Northwest China	Gansu province, Ningxia Hui Autonomous Region, Qinghai province, Shaanxi province, Xinjiang Uyghur Autonomous Region

### Statistical analysis

#### Pooling prevalence of DR in China

The crude prevalence of DR was first computed for each study and then double-arcsine transformed by using the Freeman-Tukey method [31-33]. Heterogeneity among eligible studies was assessed with the Cochran's Q statistic and *I*^2^ index (the proportion of total variability due to true between-study heterogeneity beyond chance) [34,35]. A p-value of less than 0.1 showed the presence of heterogeneity, and *I*^2^ values of less than 25% corresponded to mild heterogeneity, of from 25% to 50% reflected moderate heterogeneity, and of greater than 50% represented high heterogeneity, respectively [34-36]. Because of the substantial heterogeneity noted between individual studies, a random-effects (DerSimonian and Laird method) meta-analysis was used to adjust for variability and pool the study-specific prevalence rates [32,36]. For each meta-analysis, a leave-one-out sensitivity analysis was developed to assess the robustness of the pooled results. By removing one study at a time to run the meta-analysis without it, the sensitivity analysis could test whether single studies had a disproportionally excessive influence on the pooled results [37]. Publication bias was checked by visual inspection of funnel plots, and tested for significance with Egger’s regression test for funnel plot asymmetry and Begg’s rank correlation test [38-40]. The prevalence rates of any DR, NPDR and PDR in both general population and people with DM were pooled with this approach respectively.

### Subgroup meta-analysis and meta-regression of DR prevalence in people with DM

For the prevalence of any DR in people with DM, potential sources of heterogeneity were investigated using subgroup meta-analysis and meta-regression. In subgroup meta-analysis, the prevalence of any DR was estimated for different age groups and DM duration groups. This was done because age- and DM duration-specific prevalence of any DR in people with DM was universally provided by the included studies. Moreover, the individual associations between prevalence of any DR and study-level variables were evaluated by univariable meta-regression using unrestricted maximum likelihood method. The prespecified variables included gender (male vs female), setting (urban, rural and mixed), geographic region and study year. Because only a few variables were individually significant, a multivariable meta-regression was not subsequently performed.

### Meta-analysis of risk factors for DR in people with DM

To investigate the risk factors for any DR in people with DM, a random-effects meta-analysis was employed a priori because of anticipated variation in study populations, geography and study design. As a rule, we only included risk factors that were investigated in at least three studies using multivariate design, and the definitions of the same risk factor should be similar across all included studies. Finally, 11 factors (advanced age, male gender, DM duration, insulin treatment, fasting blood glucose [FBG], 2-hour postprandial blood glucose [2h-PBG], glycated haemoglobin A1c [HbA1c], total cholesterol [TC], triglyceride [TG], body mass index [BMI], systolic blood pressure [SBP]) met our criteria and were included in meta-analysis.

### Estimation of national and subnational burden of DR in 2010

At the final stage, the national number of cases with any DR (“any DR envelope”) was estimated by multiplying the age-specific prevalence of any DR in people with DM with the corresponding number of people with DM in China. For this purpose, the 2011 national baseline survey of China Health and Retirement Longitudinal Study (CHARLS), a nationally representative survey of Chinese people aged 45 years and older by using a four-stage, stratified, cluster sampling procedure, was selected to provide prevalence estimates of DM in China for the year 2010, and the Chinese population data were obtained from the United Nations Population Division (UNPD) [41]. The number of people with DM was estimated with the prevalence of DM and the corresponding population size. The study design and implement of CHARLS have been published previously and the procedures for deriving the prevalence of DM are outlined in Table S2 and Figures S1-S2 in **Online Supplementary Document(Online Supplementary Document)** [42,43]. Then the national number of people with any DR (“any DR envelope”) was distributed into six geographical regions in China (East China, North China, Northeast China, Northwest China, South Central China, Southwest China) by taking the effects of major risk factors on the prevalence of any DR in those regions [44,45]. Four statistically significant risk factors (advanced age, rural setting, elevated FBG level and higher HbA1c concentration) were chosen because they were all objective indicators. DM duration and insulin treatment, although being significantly associated with the prevalence of any DR, were not selected because they were highly subject to the diagnosis and treatment of DM, socioeconomic circumstances or geography, and therefore might introduce bias in our estimation of DR burden at the subnational level.

A two-sided p-value of less than 0.05 indicated statistical significance except for the Q statistics, in which a significance level of less than 0.1 was specified. All statistical analyses were done with R version 3.3.0 (R Foundation for Statistical Computing, Vienna, Austria) and STATA version 14.0 (STATA Corporation, College Station, TX, USA). All maps were drawn by ArcMap version 10.1 (Environmental Systems Research Institute, Redlands, CA, USA) using the China base map obtained from the Global Administrative Areas (GADM) database (GADM, 2015, version 2.0; www.gadm.org).

## RESULTS

### Summary of systematic review

**Figure 1:**
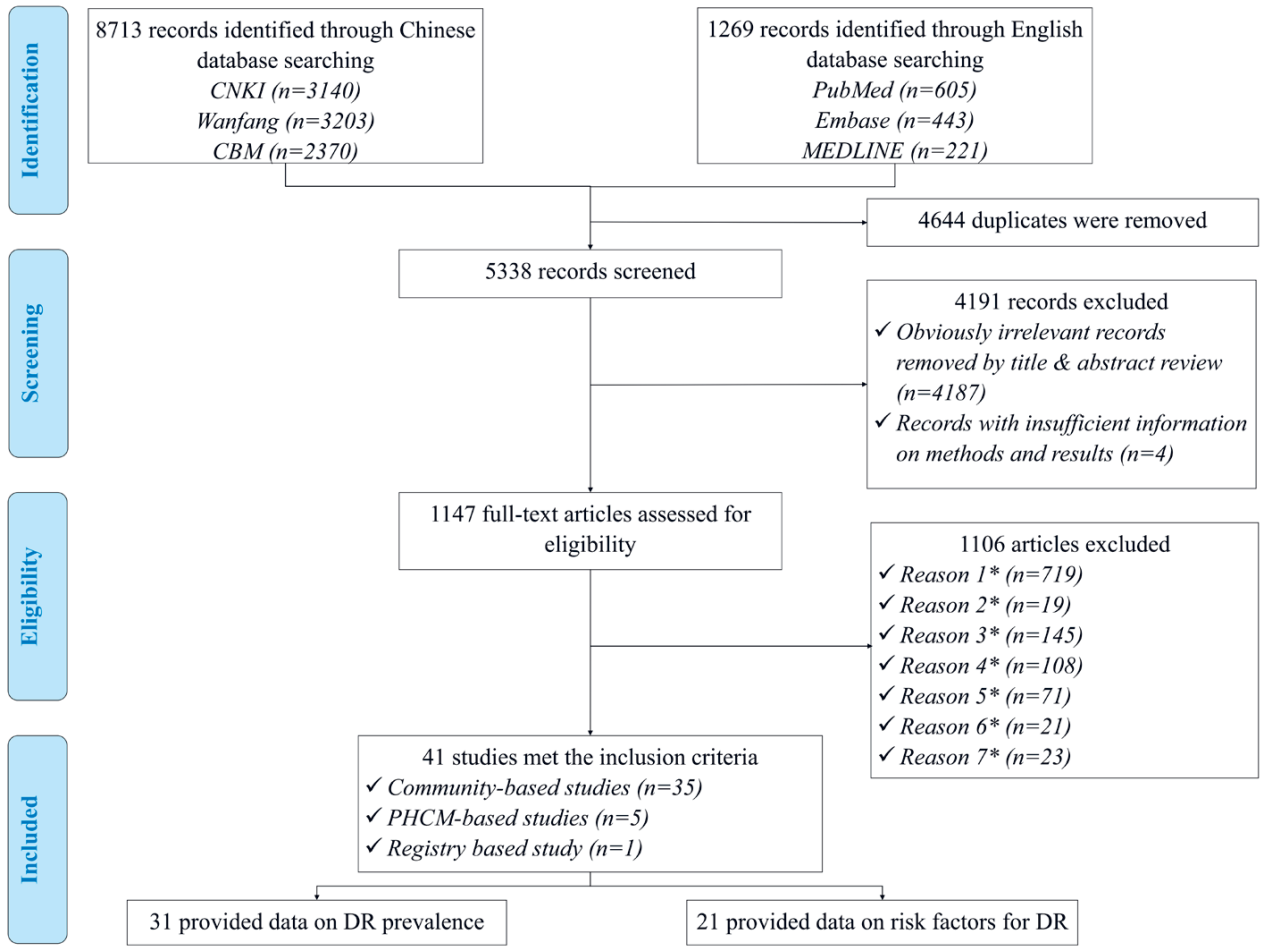
Systematic review flow diagram of studies on the prevalence of and risk factors for diabetic retinopathy (DR) in China. PHCM - Primary Health Care Management; *Reason 1 – Studies that were not community-based, PHCM-based or registry-based; *Reason 2 – Studies that were not based in China; *Reason 3 – Articles with no numerical prevalence measure of DR or didn’t report risk factor for DR in people with diabetes mellitus (DM); *Reason 4 – Studies with no clear assessment methods or grading systems of DR; *Reason 5 – Studies that were specifically conducted in people with unrepresentative characteristics (hypertensive patients, people with reduced vision, etc.); *Reason 6 – Multiple publications of the same study; *Reason 7 – Studies that didn’t include both newly detected and diagnosed DM cases.

The initial search strategy yielded 9982 records. After removal of 4644 duplicates, a total of 5338 records were reviewed for relevance by titles and abstracts, of which 1147 were assessed in full-text form. Finally, 41 articles met eligibility criteria and were included in the systematic review. Of these, 31 studies provided information on the prevalence of DR and 21 explored potential risk factors for DR. The process of study selection is summarised in **Figure 1** according to the PRISMA guidelines. A full list of the included studies is shown in Table S3 in **Online Supplementary Document(Online Supplementary Document)**.

**Figure 2:**
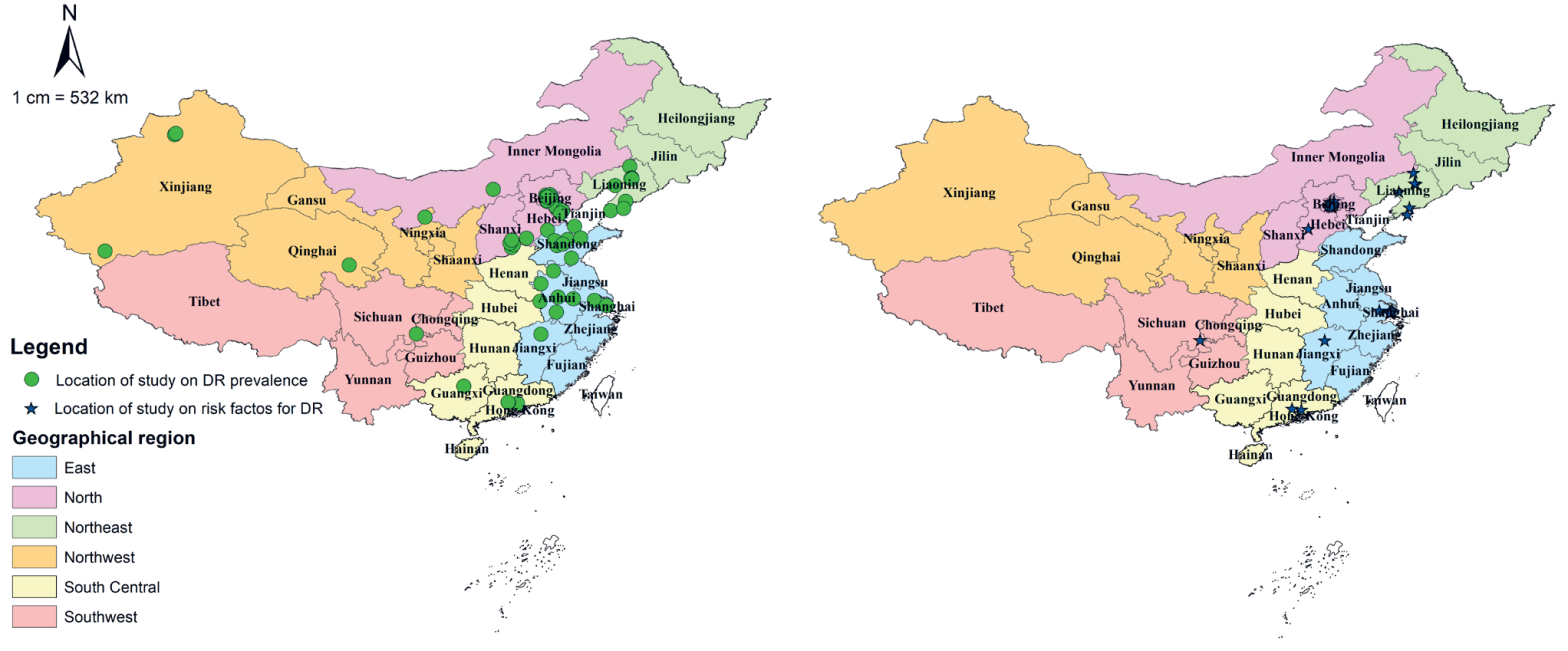
Geographical distribution of included studies on prevalence of and risk factors for diabetic retinopathy (DR) in China.

All included studies were cross-sectional in design and assessed DR by using FP. **Table 2** summarises the main characteristics of all included studies, and Table S4 in **Online Supplementary Document(Online Supplementary Document)** lists the detailed characteristics of every study. For the 31 studies that reported the prevalence of DR and the 21 studies on risk factors for DR, the majority were published after 2010, implying the necessity for an updated analysis of the epidemiology of DR in China. The studies on the prevalence of DR were all community-based investigations, covering all the six geographic regions across China (see **Figure 2**). For those on risk factors for DR, more than half were community-based (71%, n = 15), whereas more than one third were conducted in East China (38%, n = 8). There were no studies from Northwest China on which to base estimates of risk factors for DR (see **Figure 2**).

**Table 2:** Main characteristics of the included studies on the prevalence of and risk factors for diabetic retinopathy in China*

Characteristics of study	Number of studies (%)	
**Studies on DR prevalence (n = 31)**	**Studies on risk factors for DR (n = 21)**
**Year published:**		
1990-1999	4 (12.9)	1 (4.8)
2000-2009	7 (22.6)	5 (23.8)
2010-2017	20 (64.5)	15 (71.4)
**Study design:**		
Community-based	31 (100.0)	15 (71.4)
PHCM-based	0 (0.0)	5 (23.8)
Registry-based	0 (0.0)	1 (4.8)
**Setting:**		
Urban	12 (38.7)	15 (71.4)
Rural	7 (22.6)	3 (14.3)
Mixed	12 (38.7)	3 (14.3)
**Gender:**		
Mixed	13 (41.9)	3 (14.3)
Both	18 (58.1)	18 (85.7)
**Sample size of DM:**		
≤200	6 (19.4)	2 (9.5)
201-500	12 (38.7)	5 (23.8)
501–1000	9 (29.0)	6 (28.6)
>1000	4 (12.9)	8 (38.1)
**Grading system:**		
ICDRDSS	12 (38.7)	11 (52.4)
ETDRS	6 (19.4)	7 (33.3)
NOFDG	3 (9.7)	1 (4.8)
NCOFD	9 (29.0)	2 (9.5)
CBM	1 (3.2)	0 (0.0)
**Geographic regions:**		
North China	11 (35.5)	7 (33.3)
Northeast China	5 (16.1)	3 (14.3)
East China	5 (16.1)	8 (38.1)
South Central China	4 (12.9)	2 (9.5)
Southwest China	1 (3.2)	1 (4.8)
Northwest China	5 (16.1)	0 (0.0)

DR – diabetic retinopathy, DM – diabetes mellitus, PHCM – Primary Health Care Management, ICDRDSS – International Clinical Diabetic Retinopathy Disease Severity Scale, ETDRS – Early Treatment of Diabetic Retinopathy Study, NOFDG – National Ocular Fundus Diseases Group, NCOFD – National Conference on Ocular Fundus Diseases, CBM – China Medical Board

*11 studies reported both prevalence of DR and risk factors for DR, therefore the sum of the number of studies exceeded 41.

### Pooled prevalence of DR in China during 1990 and 2017

**Figure 3:**
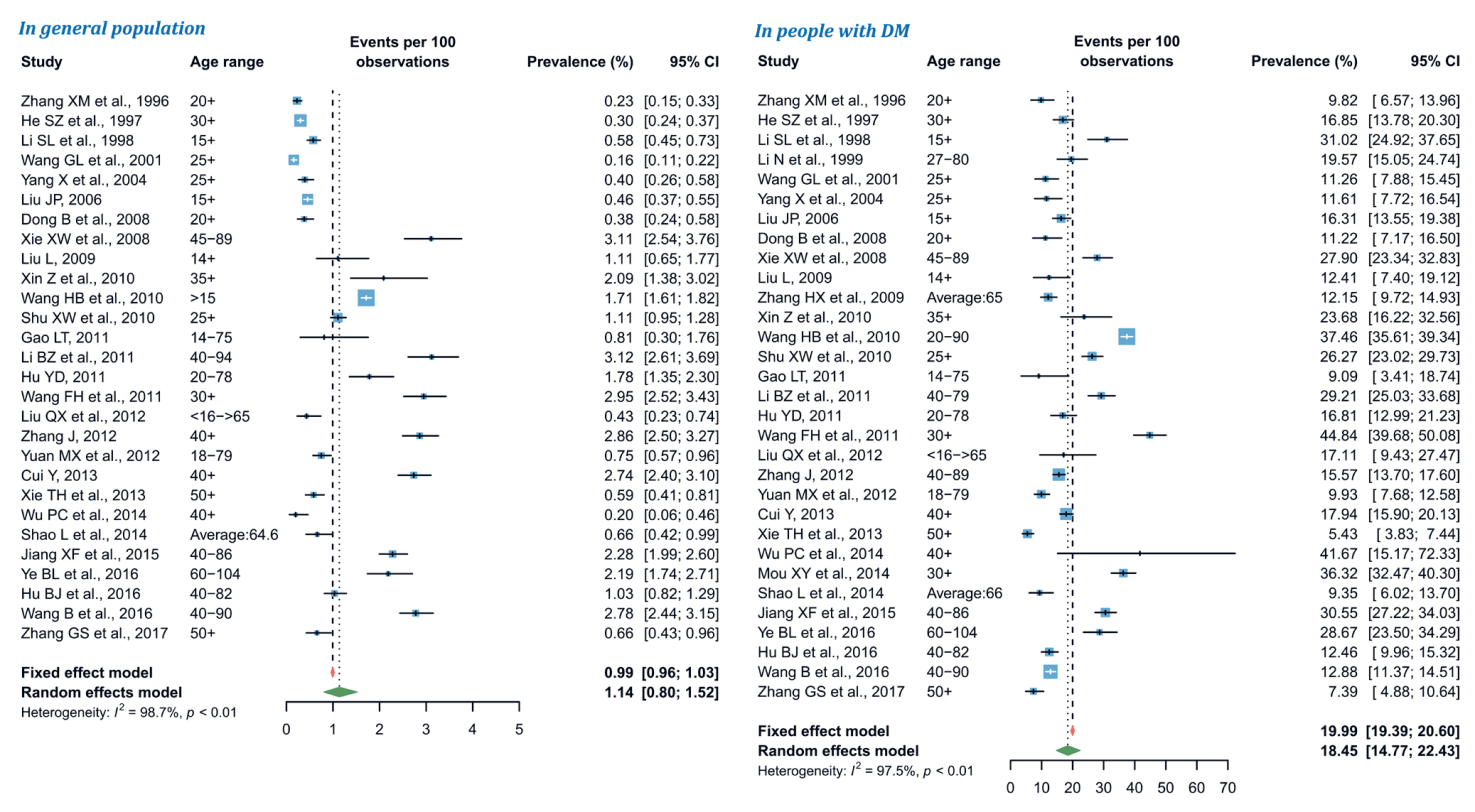
Pooled prevalence of any diabetic retinopathy (DR) in general population and in people with DM by random-effects meta-analysis. There were 28 studies for synthesizing the prevalence of any DR in general population and 31 in people with DM.

By using random-effects meta-analysis, the pooled prevalence of any DR in general Chinese population was 1.14% (95% CI = 0.80-1.52), and that in people with DM was 18.45% (95% CI = 14.77-22.43) (**Figure 3**). According to the leave-one-out sensitivity analysis (Figure S3 in **Online Supplementary Document(Online Supplementary Document)**), the pooled prevalence of any DR in general population varied from 1.08% (95% CI = 0.76-1.46) to 1.19% (95% CI = 0.86-1.58), and that in people with DM ranged from 17.67% (95% CI = 14.12-21.53) to 19.01% (95% CI = 15.38-22.94), no single study significantly influenced the overall pooled prevalence in the meta-analysis. No publication bias was evident based on the visual evaluation of the funnel plot, Egger’s test and Begg’s test (Figure S4 in **Online Supplementary Document(Online Supplementary Document)**).

**Figure 4:**
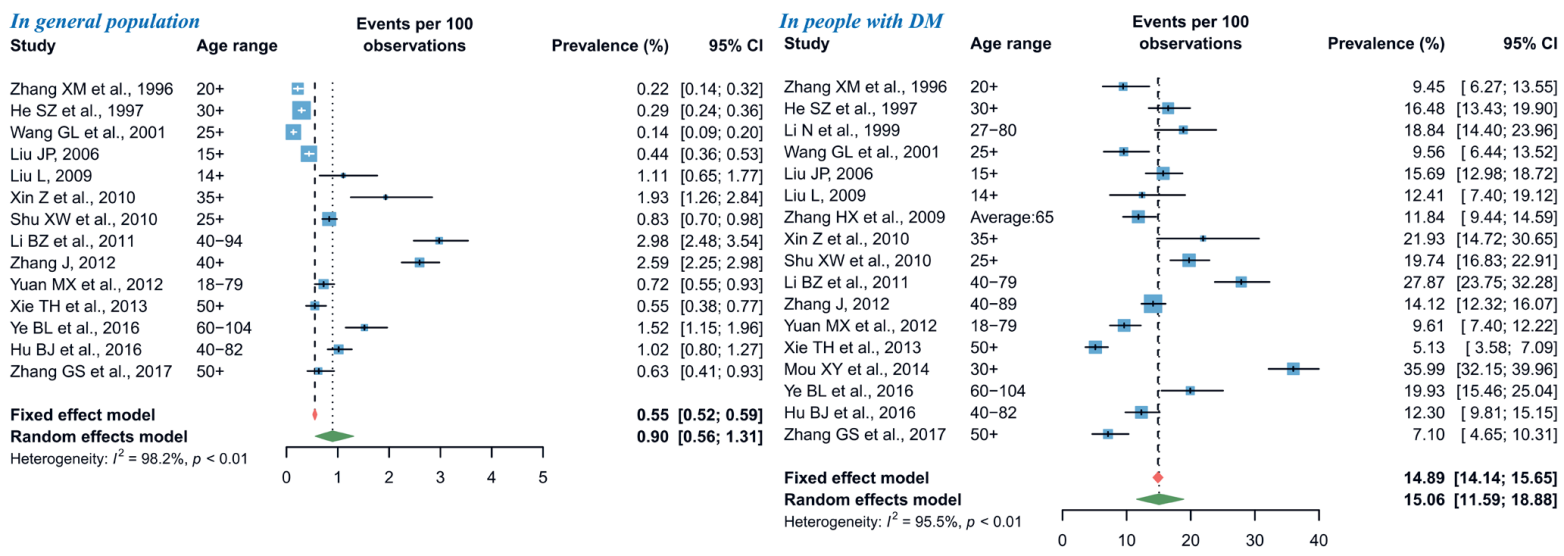
Pooled prevalence of nonproliferative diabetic retinopathy (NPDR) in general population and in people with diabetes mellitus (DM) by random-effects meta-analysis. There were 14 studies for synthesizing the prevalence of NPDR in general population and 17 in people with DM.

For NPDR, the pooled prevalence in general population was 0.90% (95% CI = 0.56-1.31), and that in people with DM was 15.06% (95% CI = 11.59-18.88) by use of random-effects meta-analysis (**Figure 4**). The leave-one-out sensitivity analysis suggested that no individual study significantly influenced the overall pooled prevalence in the meta-analysis (Figure S5 in **Online Supplementary Document(Online Supplementary Document)**), where the pooled prevalence of NPDR in general population ranged from 0.79% (95% CI = 0.49-1.14) to 0.99% (95% CI = 0.63-1.42) and that in people with DM from 13.92% (95% CI = 11.20-16.87) to 15.85% (95% CI = 12.48-19.53). Among studies that reported the prevalence of NPDR in general population, potential publication bias was revealed by the asymmetrical shape of funnel plot, Egger’s test and Begg’s test, whereas no publication bias was suggested for studies that reported the prevalence of NPDR in people with DM (Figure S6 in **Online Supplementary Document(Online Supplementary Document)**).

**Figure 5:**
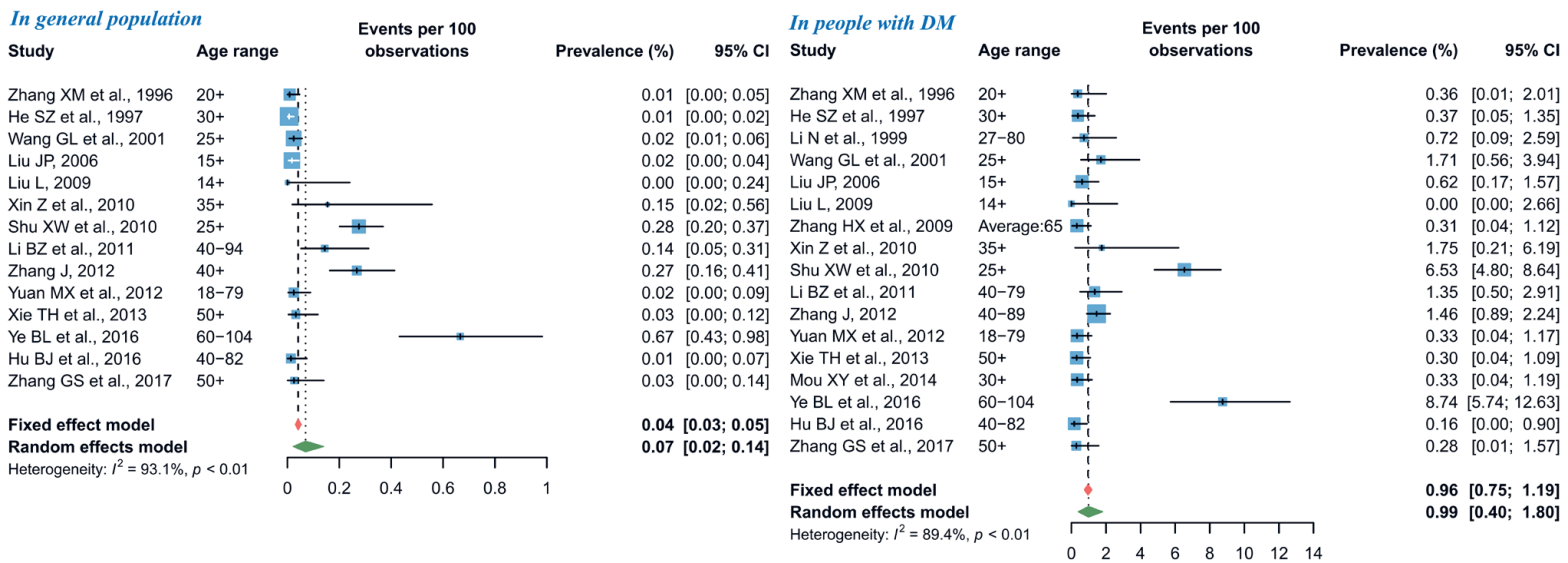
Pooled prevalence of proliferative diabetic retinopathy (PDR) in general population and in people with diabetes mellitus (DM) by random-effects meta-analysis. There were 14 studies for synthesizing the prevalence of PDR in general population and 17 in people with DM.

As shown in **Figure 5**, the pooled prevalence of PDR from random-effects meta-analysis was 0.07% (95% CI = 0.02-0.14) in general population and 0.99% (95% CI = 0.40-1.80) in people with DM. The subsequent sensitivity analysis showed that the pooled prevalence of PDR was not affected unduly by a single study, where the pooled prevalence rates ranged from 0.05% (95% CI = 0.01-0.10) to 0.08% (95% CI = 0.03-0.16) in general population, and from 0.76% (95% CI = 0.30-1.39) to 1.07% (95% CI = 0.43-1.95) in people with DM (Figure S7 in **Online Supplementary Document(Online Supplementary Document)**). For studies that reported the prevalence of PDR in general population, visual inspection of the funnel plot and Begg’s test demonstrated some evidence of significant publication bias, which was not confirmed by the Egger’s test. No publication bias was detected in the meta-analysis of PDR prevalence in people with DM (Figure S8 in **Online Supplementary Document(Online Supplementary Document)**).

### Subgroup meta-analysis and meta-regression of DR prevalence in people with DM

**Figure 6:**
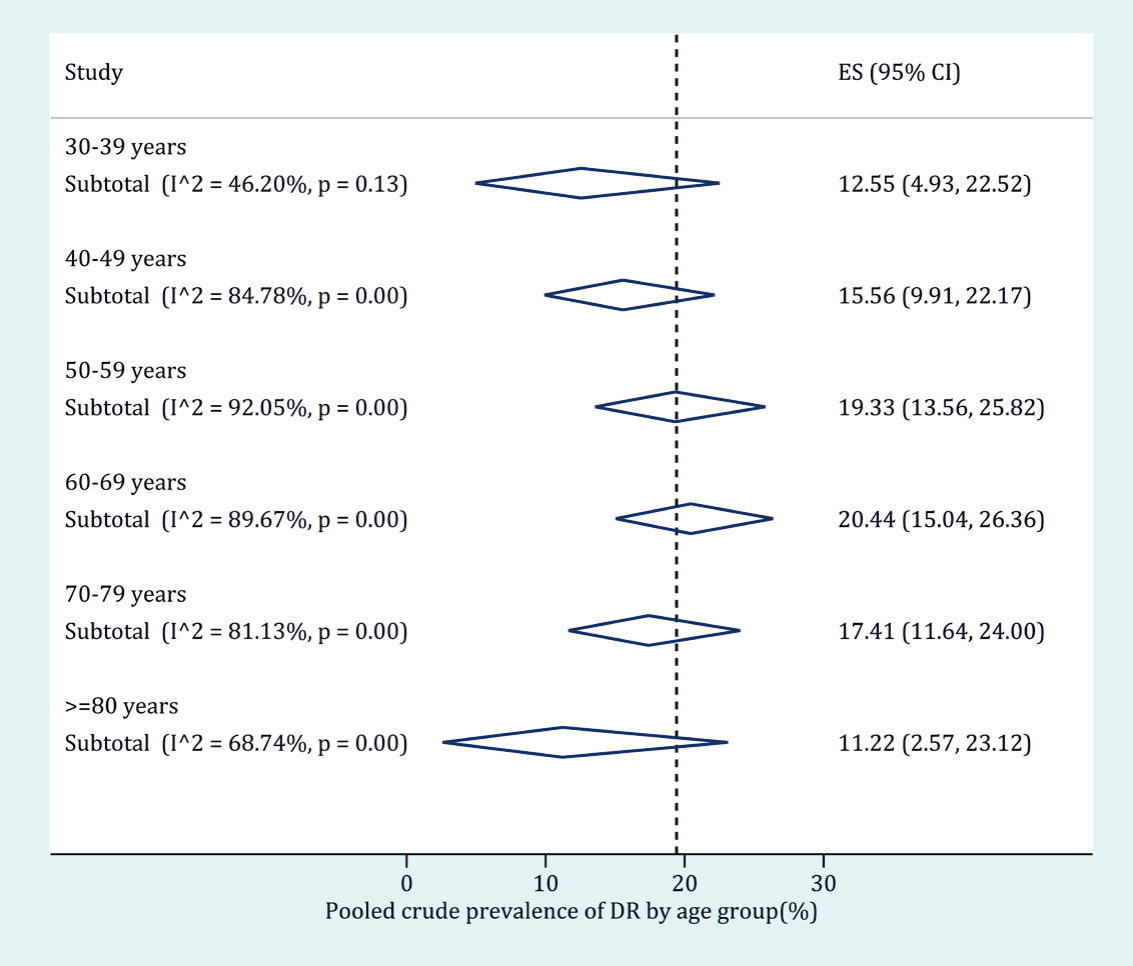
Age-specific prevalence of any diabetic retinopathy (DR) in people with diabetes mellitus (DM) by random-effects meta-analysis. The numbers of individual studies contributing to the synthesis of prevalence in each age group are 4 (for 30-39 years), 10 (for 40-49 years), 15 (for 50-59 years), 16 (for 60-69 years), 10 (for 70-79 years) and 9 (for 80-89 years) respectively.

The age-specific prevalence of any DR in people with DM was derived based on subgroup meta-analysis (**Figure 6**). The following age categories were adopted: 30-39 years, 40-49 years, 50-59 years, 60-69 years, 70-79 years and 80 years and older. Before the age of 70 years, the prevalence of any DR in people with DM kept rising from 12.55% (95% CI = 4.93-22.52) in adults aged 30-39 to 20.44% (95% CI = 15.04-26.36) in those were 60-69 years old. Then the prevalence of any DR in people with DM started to decrease, until 11.22% (95% CI = 2.57-23.12) in elderly aged 80 years and above. The detailed process of synthesizing the prevalence of any DR in people with DM in each age category can be found in Figure S9 in **Online Supplementary Document(Online Supplementary Document)**.

**Figure 7:**
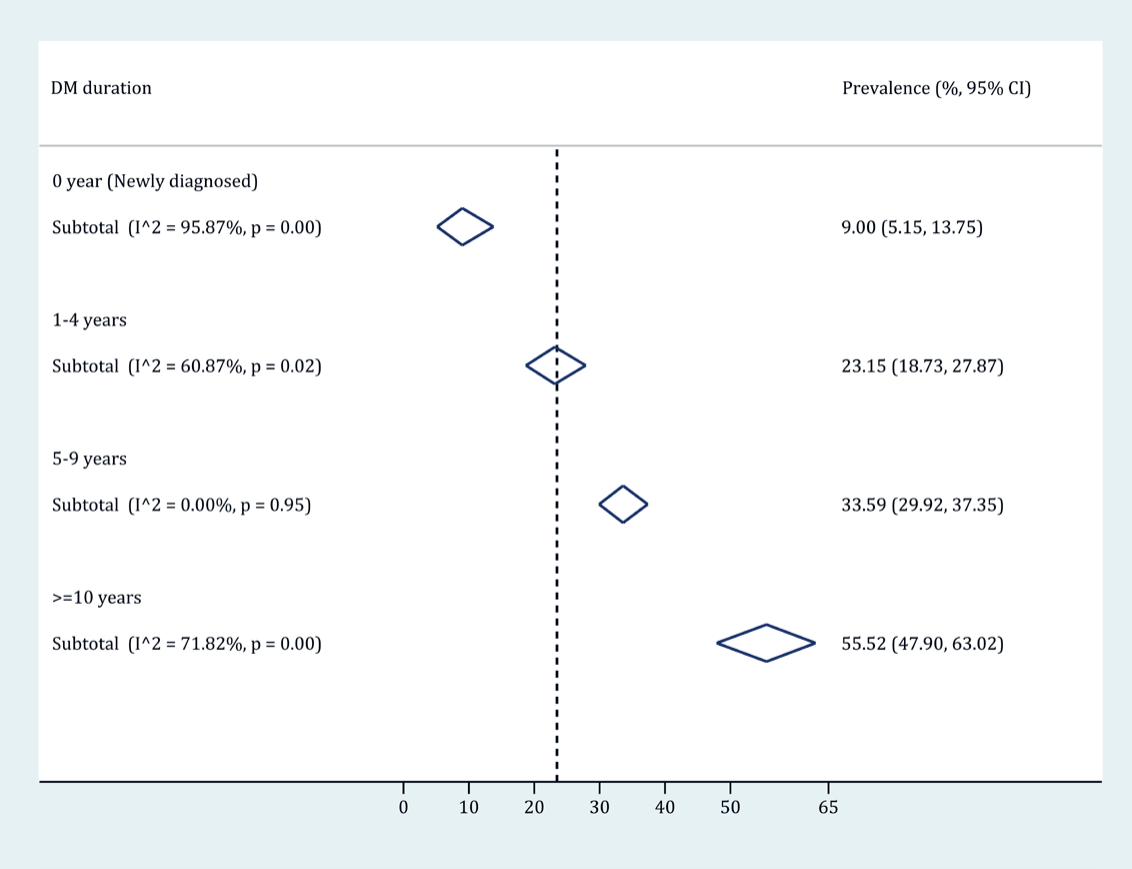
The prevalence of any diabetic retinopathy (DR) by diabetes mellitus (DM) duration group, using random-effects meta-analysis. The numbers of individual studies contributing to the synthesis of prevalence in each DM duration group are 13 (for newly diagnosed), 7 (for 1-4 years), 8 (for 5-9 years) and 9 (for ≥10 years) respectively.

By pooling the prevalence of any DR in strata of DM duration group, it was revealed that the prevalence of any DR in people with DM substantially increased with the duration of DM. Four different DM duration groups were used: 0-year (newly detected), 1-4 years, 5-9 years and 10 years and longer. According to the subgroup meta-analysis (**Figure 7**), the DM duration-specific prevalence of any DR ranged from 9.00% (95% CI = 5.15-13.75) in people with newly detected DM to 55.52% (95% CI = 47.90-63.02) in those who had been diagnosed with DM for 10 years and longer. The process of synthesizing the prevalence of any DR in each DM duration group is detailed in Figure S10 in **Online Supplementary Document(Online Supplementary Document)**.

According to the univariable meta-regression (**Table 3**), DM patients living in rural areas were more likely to have any DR than those in urban areas, with an OR of 1.22 (95% CI = 1.10-1.35). However, no evidence of gender difference, geographical variation or a secular trend in the prevalence of any DR in individuals with DM was observed.

**Table 3:** Odds ratios for any diabetic retinopathy in terms of setting, geographic region and study year from univariable meta-regression models, with 95% confidence intervals

Variable	Number of studies	OR (95% CI)	z value	*P*-value
Gender:*				
Female	18	Reference	Reference	Reference
Male		0.98 (0.88-1.08)	-0.47	0.639
**Setting:**				
Urban	12	Reference	Reference	Reference
Rural	7	1.22 (1.10-1.35)	3.74	<0.001
Mixed	12	1.01 (0.93-1.10)	0.24	0.810
**Geographic region:**				
North China	11	Reference	Reference	Reference
Northeast China	5	0.96 (0.82-1.13)	-0.48	0.632
East China	5	0.99 (0.85-1.17)	-0.07	0.946
South Central China	4	0.95 (0.80-1.13)	-0.55	0.582
Southwest China	1	0.94 (0.69-1.28)	-0.39	0.699
Northwest China	5	0.97 (0.82-1.15)	-0.34	0.735
**Study year (per decade)**	31	1.01 (0.93-1.09)	0.22	0.826

*The effect of gender was estimated based on studies where gender-specific prevalence was available.

### Synthesized effect size of risk factors for DR in people with DM

A total of 21 studies described the risk factors for any DR in people with DM by multivariate logistic regression (Table S5 in **Online Supplementary Document(Online Supplementary Document)**). Risk factors for any DR were reported in various ways, among which 11 were with consistent definitions and sufficient information, and therefore were included in evidence synthesis (**Table 4**). Advanced age was found to be negatively associated with any DR, which was partly in line with our estimates on the age-specific prevalence of any DR, where the prevalence of any DR started to decrease from 70 years onwards. In accordance with the estimated DM duration-specific prevalence of any DR in subgroup meta-analysis, longer DM duration was additionally recognised as a significant risk factor for any DR. DM patients receiving insulin treatment were almost two times more likely to have any DR than those who were not treated by insulin (OR 1.99 [95% CI = 1.34-2.95]). Moreover, elevated FBG level and higher HbA1c concentration were all identified as important risk factors for any DR, with ORs per unit increase of 1.33 (95% CI = 1.12-1.59) and 1.15 (95% CI = 1.09-1.20) respectively. Individual forest plots of meta-analyses for each risk factor can be found in Table S6 in **Online Supplementary Document(Online Supplementary Document)**.

**Table 4:** Synthesized effect size of 11 risk factors for any diabetic retinopathy in people with diabetes mellitus

Risk factor	Number of studies	OR (95% CI)	z value	*P*-value
Risk factor 1-Advanced age (per year increase)	4	0.96 (0.93-1.00)	2.26	0.024
Risk factor 2-Male	5	1.41 (0.88-2.27)	1.42	0.156
Risk factor 3-DM duration (per year increase)	12	1.09 (1.06-1.12)	5.93	<0.001
Risk factor 4-Insulin treatment	5	1.99 (1.34-2.95)	3.4	0.001
Risk factor 5-FBG (per mmol/L increase)	9	1.33 (1.12-1.59)	3.23	0.001
Risk factor 6-2h PBG (per mmol/L increase)	3	1.94 (0.81-4.65)	1.48	0.138
Risk factor 7-HbA1c (per % increase)	7	1.15 (1.09-1.20)	5.80	<0.001
Risk factor 8-TC (per mmol/L increase)	3	0.97 (0.78-1.20)	0.32	0.749
Risk factor 9-TG (per mmol/L increase)	3	1.66 (0.74-3.73)	1.24	0.216
Risk factor 10-BMI (per kg/m^2^ increase)	6	1.07 (0.94-1.21)	1.06	0.289
Risk factor 11-SBP (per mmHg increase)	5	1.03 (1.00-1.07)	1.96	0.05

OR – odds ratio, CI – confidence interval, DM – diabetes mellitus, FBG – fasting blood glucose, PBG – postprandial blood glucose, HbA1c – glycated haemoglobin A1c,TC – total cholesterol, TG – triglyceride, BMI – body mass index, SBP – systolic blood pressure

### National and subnational number of people with DR in 2010

**Figure 8:**
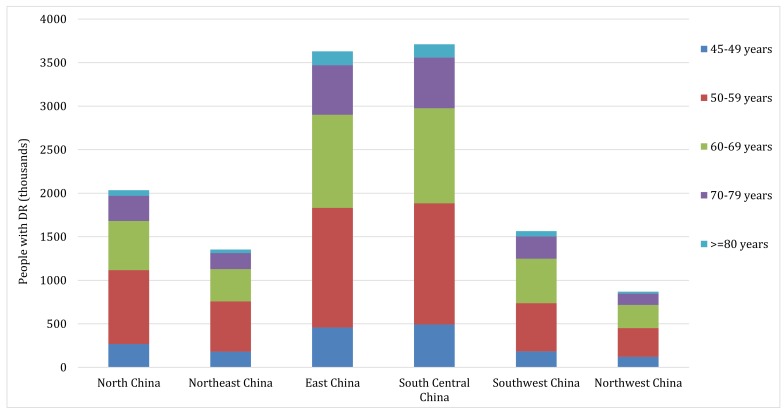
Estimated regional number of middle-aged and older Chinese with any diabetic retinopathy (DR) and contributing age groups in 2010.

According to the CHARLS 2011, the weighted prevalence of DM was 17.22% (95% CI = 15.57-19.00) in middle-aged and older Chinese in 2010 (see Table S7 in **Online Supplementary Document(Online Supplementary Document)** for more details). By applying the age-specific prevalence of any DR in people with DM and the corresponding age-specific DM cases, the number of middle-aged and older Chinese with any DR was estimated to be 13.16 million (95% CI = 8.95-18.00) in 2010, translating to an overall prevalence of 3.06% (95% CI = 2.08-4.19) in general middle-aged and older Chinese and of 18.24% (95% CI = 12.41-24.95) in middle-aged and older Chinese with DM (**Table 5**). Based on the variations of population age structure, setting, mean FBG and mean HbA1c levels, the national DR cases were distributed into six geographic regions. As illustrated in **Table 5** and **Figure 8**, South Central China harboured the most DR cases (3.71 million [95% CI = 2.52-5.09]), while Northwest China had the least (0.87 million [95% CI = 0.60-1.18]). Regarding the prevalence of any DR at regional level, it was estimated that the prevalence of any DR in general middle-aged and older Chinese was the highest in North China (3.76% [95% CI = 2.56-5.12]) and the lowest in Southwest China (2.55% [95% CI = 1.74-3.48]); For the prevalence of any DR in middle-aged and older Chinese with DM, it was the highest in Northwest China, while the lowest in East China (17.67% [95% CI = 11.97-24.24]).

**Table 5:** Estimated prevalence and number of middle-aged and older Chinese with any diabetic retinopathy in China in 2010, by geographic region

Region	Prevalence of any DR in general people (%, 95% CI)	Prevalence of any DR in people with DM (%, 95% CI)	Number of people with DR (million, 95% CI)
North China	3.76 (2.56-5.12)	18.54 (12.66-25.28)	2.03 (1.39-2.77)
Northeast China	3.22 (2.20-4.39)	18.93 (12.94-25.81)	1.35 (0.92-1.84)
East China	2.74 (1.86-3.76)	17.67 (11.97-24.24)	3.63 (2.46-4.98)
South Central China	3.31 (2.25-4.54)	17.97 (12.19-24.63)	3.71 (2.52-5.09)
Southwest China	2.55 (1.74-3.48)	18.73 (12.76-25.59)	1.56 (1.07-2.14)
Northwest China	3.09 (2.12-4.20)	19.39 (13.30-26.35)	0.87 (0.60-1.18)
China	3.06 (2.08-4.19)	18.24 (12.41-24.95)	13.16 (8.95-18.00)

DR – diabetic retinopathy, DM – diabetes mellitus, CI – confidence interval

## DISCUSSION

By combining all available epidemiological data on the prevalence of DR in China from 1990 onwards, we estimated that in general population, the pooled prevalence of any DR, NPDR and PDR was 1.14% (95% CI = 0.80-1.52), 0.90% (95% CI = 0.56-1.31) and 0.07% (95% CI = 0.02-0.14); In people with DM, the pooled prevalence rates were 18.45% (95% CI = 14.77-22.43), 15.06% (95% CI = 11.59-18.88) and 0.99% (95% CI = 0.40-1.80) for any DR, NPDR and PDR, respectively. The prevalence of any DR in DM patients peaked between 60 and 69 years of age, and increased steeply with the duration of DM. DM patients residing in rural China were at a higher risk to have any DR than those in urban areas. In addition, insulin treatment, elevated FBG level and higher HbA1c concentration were confirmed to be associated with a higher prevalence of any DR in people with DM. In 2010, a total of 13.16 million (95% CI = 8.95-18.00) Chinese aged 45 years and above were living with any DR, among whom the most were in South Central China and the least were in Northwest China. Collectively, these data suggest a considerable burden of DR in China.

To the best of our knowledge, this systematic review and meta-analysis provides a comprehensive estimation of the prevalence, risk factors and burden of DR in China. Although this study is subsequent to the first synthesized analysis by Liu and colleagues, many new merits are highlighted [22]. The principal strengths of this study include a comprehensive search strategy in both Chinese and English databases and a dual review process, which increased our ability to capture all studies on DR epidemiology in China. Ultimately, our estimation of DR prevalence was based on a total of 31 studies, which was more than 1.5 times the number of studies included in the first systematic review on DR in China. Another important feature that distinguished our study with the previous systematic review by Liu and colleagues was that only community-based studies incorporating both newly detected and already diagnosed DM cases were included for the estimation of DR prevalence, therefore the representativeness of our estimates can be greatly guaranteed. Regarding risk factors for DR, only studies that provided estimates of OR using multivariate study designs were included, therefore community-based, PHCM-based and registry-based studies could all contribute, ensuring a sufficient power for conducting reliable synthesized assessments. Furthermore, the definitions of risk factors in included studies were similar, as well as in the CHARLS 2011 [42,43]. Before pooling, an arcsine transformation was conducted to stabilise the variance of prevalence rates, which reduced the bias associated with small and large prevalence values on the pooled estimates to a large extent [32,33]. Although differences existed in the prevalence rates of DR across different subgroups, our detailed assessment of any DR prevalence by age and DM duration group, and identification of risk factors for any DR could serve as a source of primary information and guide policy making and rational planning of health services, especially in areas where local investigations on the epidemiology of DR are absent.

Before interpreting the findings, potential limitations of this systematic review and meta-analysis should be carefully considered. First, the pooled prevalence of NPDR and PDR in general population might have been affected by publication bias. Generally, publication bias arises because statistically significant results are more likely to be published than non-significant results, combining these studies for analysis could, therefore, introduce bias [40,46,47]. Unfortunately, we could not completely rule out publication bias because of the observational nature of our study. Second, there are inherent disadvantages in pooling prevalence form disparate studies. Due to the absence of stratified prevalence data for NPDR and PDR, we were not able to further explore sources of heterogeneity by subgroup meta-analysis and meta-regression for these two subtypes of DR. For any DR, sufficient data were available to pool the prevalence estimates and no publication was detected. However, our subgroup analysis on the prevalence of any DR by age group and DM duration group was only based on a limited number of studies that provided correspondingly stratified prevalence estimates. Third, only 11 risk factors with similar definitions across the included studies were systematically assessed, among which advanced age, longer DM duration, insulin treatment, elevated FBG level and higher HbA1c concentration were identified to be associated with a higher prevalence of any DR. However, because of the paucity of reported ORs, the effects of TC, TG and SBP should be further confirmed with new data coming in from future studies. In addition, previous studies have suggested that socioeconomic factors, including the availability and costs of DM management, were also likely to contribute to the disparities in DM severity and DR prevalence rates in different subgroups, but could not to be assessed in the current study [1,2,9]. Fourth, the number of DM cases in China for generating the national and subnational burden of any DR was derived from the CHARLS 2011, which was nationally representative but only conducted in middle-aged and older population [42,43]. Therefore, the estimated number of people with any DR in this study was only for people aged 45 years and above. When distributing the national DR cases into the six geographic regions, we only took the effects of four objective indicators into account, namely, advanced age, rural setting, elevated FBG level and higher HbA1c concentration. Other subjective risk factors (eg, insulin treatment and DM duration) and potential factors that might be associated with the prevalence of DR were not included in our analysis of regional burden of DR in China, which might reduce the reliability of our estimation at the subnational level. Bearing those limitations in mind, the results presented in this study should be interpreted judiciously.

In this study, significant heterogeneity was noted in pooling the prevalence rates of DR. The main sources of heterogeneity in the included studies pertained to the different characteristics of study population. After omitting each study at a time, the pooled prevalence of any DR was robust and consistent. The pooled prevalence of any DR in Chinese people with DM was lower in our study than that in the global DR study (18.45% vs 25.08%) [2]. Given that the estimated prevalence of any DR in Chinese people with DM presented in the global DR study was based on studies that were conducted both within and outside China, any differences in exposure levels of risk factors might explain this discrepancy. Compared with the pooled prevalence of any DR in Chinese people with DM reported by Liu and colleagues, our study revealed a relatively lower prevalence rate (23.0% vs 18.45%) [22]. There are a number of possible reasons for this difference. First, the improvement of primary health care management in China might have resulted in a lower incidence of DR in recent years. Second, more recent investigations might include more newly detected DM patients. The incidence of DM is higher than that of DR, resulting in a relatively larger denominator for calculating the prevalence of DR. Most importantly, individual studies included in the systematic review and meta-analysis by Liu and colleagues were not solely focused on generally Chinese population, where both newly detected and diagnosed DM (self-reported physician diagnosed DM in some studies) cases should exist simultaneously. Two individual studies that were conducted in people with diagnosed DM were included in their final synthesis, the erroneous omission of people with newly detected or early-stage DM from the sample denominator would, therefore, lead to an overestimation of DR prevalence [22]. Furthermore, a study included in their synthesized analysis was specifically conducted in a group of people with higher risk for pre-diabetes (eg, people with familial DM history, hypertension, overweight/obesity, dyslipidemia, cardiovascular disease /stroke or a gestational history of large babies [for women]) rather than in general population, which will also add further possibility of an overestimation [22,48].

In our analysis, the prevalence of any DR was found to peak between 60 and 69 years of age, which is in line with the age-specific prevalence estimates of DR among Americans [4,49]. In elderly with DM, the incidence of DR is relatively lower than that in younger people [9,50]. Given that DR is a marker for severe DM and other life-threatening complications, a reduced survival rate has been observed in older people living with DR [51-55]. Therefore, this pattern of declining DR prevalence in elderly seems to be driven by the combination of reduced incidence and improved mortality. Unsurprisingly, the prevalence of DR is strongly associated with the duration of DM, which has been validated by both sub-group meta-analysis and our meta-analysis on major risk factors for DR in this present study. This finding is consistent with other previous investigations and synthesized analyses [2,55,56]. As revealed by our analysis, more than half of all patients with DM for 10 years and longer will develop some degree of DR, underscoring the importance of optimal management of DM and early detection of DM complications in those living with DM. In this study, we noticed a higher prevalence of DR in DM patients living in rural China than that in those living in urban areas. This urban-rural disparity of DR prevalence in people with DM is in line with the study by Liu and colleagues [22]. In the Chinese context, awareness (a history of physician-diagnosed), treatment (proportion of individuals taking diabetes medications), and control (the proportion of individuals with an HbA1c concentration of less than 7.0%) of DM among rural dwellers are all lower than that in urban dwellers, partly due to lower economic development level and restricted primary health resources in rural China [23,51,57]. The delayed diagnosis and non-optimal management of DM might be the primary causes of a higher prevalence of DR in rural China, but still need further confirmation in future studies.

Good glycemic control has long been recognized as one important factor for reducing vascular complications of DM, and it is also important in the prevention of DR [16,58]. In this study, higher levels of FBG and HbA1c have both been suggested as risk factors for DR in people with DM, which is in line with many previous investigations and synthesized results [56,59]. In addition, insulin treatment was identified to be with a higher odds of DR in DM patients according to our meta-analysis. Herein insulin treatment should not be simply concluded as a “bad treatment” which directly causes DR. In previous studies, it was suggested that a larger proportion of participants using insulin therapy were those with T1DM or with longer-duration of DM, and people with DR may have already been preferentially treated with insulin therapy [56,60,61]. In previous studies, higher SBP has been suggested as a risk factor for DR [2,58]. However, our meta-analysis of risk factors for DR only showed a slightly significant association between elevated SBP and DR. Given the effect of SBP on DR was only assessed based on five individual studies in our synthesized analysis, the lack of sufficient evidence logically calls for an updated analysis to better understand the role of SBP in the development of DR with new data coming in.

The increasing burden of DR might bring a higher pressure on available infrastructure and resources. Ideally, periodic eye examinations should be conducted by all patients with DM. Regular follow-up to detect significant retinopathy, together with prompt interventions when necessary, is believed to be the most effective method to reduce potential DR-related visual disabilities [1,3,62]. In China, screening of DR has not been well established into the primary health care system, and the need for adequate DR eye care remains largely unaddressed [63,64]. Generally, DR screening could be evaluated in office or through telemedicine, and the latter has been suggested to be accurate and more cost-effective [65-67]. Furthermore, with the development of technology, a wholly automated approach with the assistance of artificial intelligence might be especially beneficial in under-developed areas. Even in established screening centres, those techniques also have a potential to substantially reduce the grading workload [68,69].

With new epidemiological investigations emerging, the results of this study should be updated in a timely and regular manner. In addition, there remains a genuine need for prompting international standardized DR classification systems in Chinese scientific society, to facilitate communication and comparison across the world.

## CONCLUSIONS

To conclude, this contemporary systematic review and meta-analysis estimated the prevalence, risk factors and burden of DR in China. The results from this study revealed a substantial burden of DR in China. Optimal screening of and interventions on DR should be implemented in the Chinese health system. Improved epidemiological studies on DR are still required to guide eye care programmes in China.
