# Supplementary material for: Prevalence, risk factors and burden of diabetic retinopathy in China: a systematic review and meta-analysis
Source: J Glob Health. 2018 Jun 9;8(1):010803. doi: 10.7189/jogh.08.010803 (PMC5997368; doi:10.7189/jogh.08.010803)
Supplement: Online Supplementary Document [file jogh-08-010803-s001.pdf]

Online Supplementary Document

Song et al. Prevalence, risk factors and burden of diabetic retinopathy in China: a systematic review and meta-analysis

J Glob Health 2018;8:010803

Table S1. Search strategy to identify studies reporting the prevalence of and risk factors for DR in China

CNKI

Access Date: 05 Dec 2017

Subject category: Medicine & Public Health

Sub-database: Journal, Featured journal, Doctoral dissertation, Master dissertation

检索表达式：

(SU % '糖尿病') AND (SU % '视网膜') AND (SU % '发病率' + '发生率' + '患病率'+ '罹患率' + '现患率'+ '死亡率' + '病死率'+ '流行' + '负担'+ '现况调查'+ '现况研究')

发表时间：从 1990-01-01 到 2017-12-05

Search Terms: (SU % 'tangniaobing') AND (SU % 'shiwangmo') AND (SU % 'fabinglv' + 'fashenglv' + 'huanbinglv'+ 'lihuanlv' + 'xianhuanlv'+ 'siwanglv' + 'bingsilv'+ 'liuxing' + 'fudan'+ 'xiankuangdiaocha'+ 'xiankuangyanjiu')

Published time：From 01/01/1990 to 05/12/2017

Wanfang

Access Date: 04 Dec 2017

Sub-database: Journal articles, Dissertations

检索表达式：(主题:(糖尿病)) * (主题:(视网膜)) * (主题:(发病率) + 主题:(发生率) + 主题:(患病率) +主题:(罹患率) + 主题:(现患率) + 主题:(死亡率) +主题:(病死率) + 主题:(流行) + 主题:(负担)+ 主题:(现况调查) + 主题:(现况研究))

时间：1990-2017

Search Terms: (subject: (tangniaobing))* (subject: (shiwangmo))* (subject: (fabinglv) + subject: (fashenglv) + subject: (huanbinglv)+ subject: (lihuanlv) + subject: (xianhuanlv) + subject: (siwanglv)+ subject: (bingsilv) + subject: (liuxing) + subject: (fudan)+ subject: (xiankuangdiaocha) + subject: (xiankuangyanjiu))

Date: 1990-2017

CBM-SinoMed

Access Date: 04 Dec 2017

Journal category: All journals

检索表达式：

(糖尿病) AND (视网膜) AND (发病率 or 发生率 or 患病率 or 罹患率 or 现患率 or 死亡率 or 病死率 or 流行 or 负担 or 现况调查 or 现况研究)

时间：1990-2017

Search Terms: ((tangniaobing))* ((shiwangmo))* ((fabinglv) OR (fashenglv) OR (huanbinglv) OR (lihuanlv) OR (xianhuanlv) OR (siwanglv) OR (bingsilv) OR (liuxing) OR (fudan) OR (xiankuangdiaocha) OR (xiankuangyanjiu))

Date: 1990-2017

PubMed

Access Date: 04 Dec 2017

Search Terms:

((diabetic retinopathy) AND (China OR Chinese OR Hongkong OR Macau OR Taiwan) AND (inciden* OR prevalen* OR morbidity OR mortality OR epidemiology)) AND ("1990/01/01"[Date - Publication] : "2017/12/04"[Date - Publication])

Embase (Ovid)

Access Date: 04 Dec 2017

#	Searches
**1**	diabetic retinopathy.mp. or exp diabetic retinopathy/
**2**	China.mp. or exp China/
**3**	exp Chinese/ or Chinese.mp.
**4**	Hong Kong.mp. or exp Hong Kong/
**5**	Macau.mp. or exp Macao/
**6**	Taiwan.mp. or exp Taiwan/
**7**	exp incidence/ or inciden*.mp.
**8**	exp prevalence/ or prevalen*.mp.
**9**	morbidity.mp. or exp morbidity/

**10**	exp mortality/ or Mortality.mp.
**11**	exp epidemiology/ or Epidemiology.mp.
**12**	2 or 3 or 4 or 5 or 6
**13**	7 or 8 or 9 or 10 or 11
**14**	1 and 12 and 13
**15**	limit 14 to yr="1990 -Current"

Medline (Ovid)

Access Date: 04 Dec 2017

Search Terms:

#	Searches
**1**	diabetic retinopathy.mp. or exp Diabetic Retinopathy/
**2**	China.mp. or exp China/
**3**	Chinese.mp.
**4**	Hong Kong.mp. or exp Hong Kong/
**5**	Macau.mp. or exp Macau/
**6**	Taiwan.mp. or exp Taiwan/
**7**	exp Incidence/ or inciden*.mp.
**8**	exp Prevalence/ or prevalen*.mp.
**9**	Morbidity.mp. or exp Morbidity/
**10**	Mortality.mp. or exp Mortality/
**11**	Epidemiology.mp. or exp Epidemiology/
**12**	2 or 3 or 4 or 5 or 6
**13**	7 or 8 or 9 or 10 or 11
**14**	1 and 12 and 13
**15**	limit 14 to yr="1990 -Current"

Table S2. Description of the China Health and Retirement Longitudinal Study (CHARLS) 2011

<table>
<tr><td>Study design and sampling procedure</td><td>A four-stage, stratified, cluster sampling procedure was used to draw eligible samples in CHARLS 2011: First, all counties in 28 provinces of China (except Tibet province, Hainan province and Ningxia Hui Autonomous Region) were stratified by geographic region, setting (urban and rural) and economic level (per capita statistics on the gross domestic product). Among them, 150 counties were randomly chosen by a probability-proportional-to-size (PPS) sampling technique. Second, three primary sampling units (PSU, communities in urban areas or administrative villages in rural areas) were randomly selected from each of those 150 counties. Third, all the dwellings within the 450 chosen PSU were outlined on Google Earth maps using the specifically designed “CHARLS-GIS” software, among which at least 24 households were randomly selected. Fourth, in the selected households, if there were more than one member aged 45 years, one such member was randomly selected, and his/her spouse was also interviewed.</td></tr>
<tr><td>Study location</td><td>
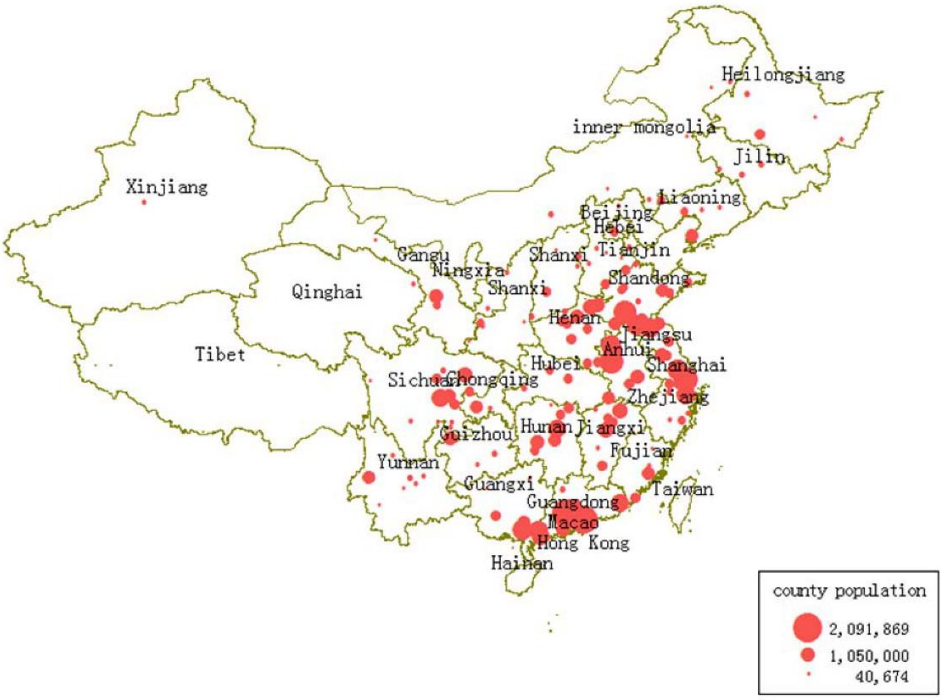

Figure S1. Geographic distribution of the sampled counties in CHARLS 2011</td></tr>
<tr><td>Procedure for deriving the prevalence of DM</td><td>Overall, the household response rate was 80.5%, and 17,708 individual participants in 10,257 households successfully completed at least one module of the survey. In the household interview, the participants’ information on demographics (i.e. age, sex, residence), socioeconomic status (i.e. educational attainment, marital status, income and expenditure), medical history and health-related behaviours (i.e. smoking, drinking) was colleated by a structured questionnaire. Among all participants, 13978 (78.9%) took part in anthropometric and physical-performance module and 11847 (66.9%) provided venous blood samples (8ml each individual). DM was defined as a self-reported physician-diagnosed diabetes, or a fasting blood glucose (FBG) ⩾126 mg/dl, or a random blood glucose ⩾200 mg/dl, or a haemoglobin A1c (HbA1c) concentration of 6.5% or above, or currently taking antidiabetic medications. People who had already been diagnosed with DM or were taking antidiabetic medications were classified as the diagnosed DM phynotype, and those who were not with</td></tr>
</table>

diagnosed DM before the investigation but had a FBG $\geqslant$126 mg/dl, or a random blood glucose $\geqslant$200 mg/dl, or a HbA1c concentration of 6.5% or abovev were classified as the newly detected DM phynotype. All calculations in CHARLS 2011 took into account the complex survey design and adopted the blood weight with household and individual response adjustment. The weighted mean FBG, mean HbA1c and prevalence of DM were strafied by age, gender, setting and geographic region. The flow diagram for selecting subjects in DM prevalence analysis is shown below:

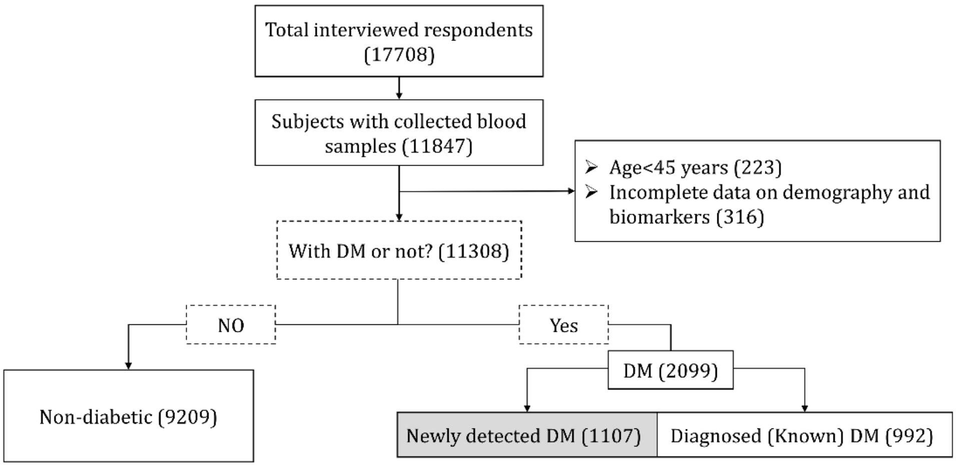

Figure S2. Flow chart for subjects included in the analysis of DM prevalence

Table S3. Full list of the included studies on the prevalence of and risk factors for DR in China (n=41)

Study ID	Reference
Community-based studies	
**DR_C1**	Xiao-mei Zhang, Shi-hong Hu. 张晓湄, 胡世红. Diabetes retinopathy detected in a population in Liuzhou municipal during epidemiologic survey of diabetes mellitus (柳州市人群糖尿病调查中视网膜病变检出情况)[J]. Journal of Clinical Ophthalmology (临床眼科杂志). 1996(04):244-5.
**DR_C2**	Shou-zhi He, Yu-luan Guo, Zhao-hui Li, et al. 何守志, 郭玉銮, 李朝辉, et al. Epidemiologic study of diabetic retinopathy in Capital Steel Company (首钢职工糖尿病视网膜病变流行病学调查)[J]. Chin J Ophthalmol (中华眼科杂志). 1997(05):62-4.
**DR_C3**	Shou-ling Li, Yan-feng Zhou, Ti Chen, et al. 李寿玲, 周艳枫, 陈逖, 杨明功, 朱美玲. Epidemiologic study about the related factors of diabetic retinopathy (糖尿病视网膜病变相关因素的流行病学调查)[J]. Chin J Ocul Fundus Dis (中华眼底病杂志). 1998(02):58-60.
**DR_C4**	Nong Li, Li-jun Ao, Shou-jun Meng, et al. 李农, 敖丽君, 孟寿军, 王华权, 杨媛魁, 许道盛. An analysis of risk factors of diabetic retinopathy in type 2 diabetic patients* (2 型糖尿病患者并发视网膜病变危险因素的分析)[J]. West China Medical Journal (华西医学). 1999(03):304-5.
**DR_C5**	Guang-lu Wang, Feng Zhang, Shen-yuan Yuan, et al. 王光璐, 张风, 袁申元, et al. A screening survey of diabetic retinopathy and other chronic complications in Beijing district (北京地区糖尿病视网膜病变及其他慢性并发症的调查)[J]. Ophthalmol CHN (眼科). 2001(03):180-2.
**DR_C6**	Xin Yang, Li-li Zhou, Ying-chun Zhao, et al. 阳新, 周黎黎, 赵迎春, 刘光英, 陈红. The relation of obesity, diabetes and vascular complications* (肥胖与糖尿病、血管并发症的关系)[J]. Liaoning Journal of Practical Diabetology (辽宁实用糖尿病杂志). 2004(03):38-9.
**DR_C7**	Ju-ping Liu. 刘巨平. Epidemiologic study of diabetic retinopathy in type 2 diabetes mellitus in Tianjin (天津市 2 型糖尿病患者糖尿病视网膜病变流行病学调查)[D]. Tianjin Medical University (天津医科大学). 2006.
**DR_C8**	Hai-ying Hu, Bin Lu, Zhao-yun Zhang, et al. 胡海英, 鹿斌, 张朝云, et al. An epidemiological study on diabetic retinopathy among type 2 diabetic patients in Shanghai (上海市中心城区 2 型糖尿病患者视网膜病变现况调查)[J]. Chin J Epidemiol (中华流行病学杂志). 2007(09):838-40.
**DR_C9**	Bin Dong, Xiang-wen Yang, Hong-juan Li, et al. 董斌, 阳湘文, 黎红娟, 周恩林. An analysis of the epidemiology and risk factors of diabetic retinopathy in communities* (社区糖尿病性视网膜病变流行病学调查及相关因素分析)[J]. Chinese Community doctors (中国社区医师(医学专业半月刊)). 2008(14):202.
**DR_C10**	Lei Liu. 刘磊. Prevalence rate and risk factors of diabetic retinopathy in Shenyang Dadong district (沈阳大东区糖尿病视网膜病变患病率及相关危险因素)[D].China Medical University (中国医科大学). 2009.
**DR_C11**	Hong-xia Zhang, Li-li Jia, Xu-hong Hou, et al. 张红霞, 贾丽丽, 侯旭宏, et al. Prevalence of and risk factors associated with diabetic retinopathy in pre-diabetic and diabetic population in Shanghai community (上海社区糖尿病前期及糖尿病人群视网膜病变患病率及相关危险因素分析)[J]. Natl Med J China (中华医学杂志). 2009;89(25):1749-52.
**DR_C12**	Zhong Xin, Ya-hong Ma, Lei Zhao, et al. 信中, 马亚红, 赵蕾, 卢毅, 石敬, 杨金奎. Prevalence and risk factors of diabetic retinopathy in rural population of Beijing (北京农村地区高血糖人群糖尿病视网膜病变患病率及危险因素分析)[J]. Clinical Focus (临床荟萃). 2010(08):672-5.

**DR_C13**	Hong-bo Wang, Feng-xian Sun, Qin Zhang, et al. 王红波, 孙凤仙, 张勤, 翟敏, 王素芳, 卢海. Epidemiologic study on the prevalence rate and risk factors of diabetic retinopathy in eastern countryside of Changzhi (山西省长治东部农村地区糖尿病视网膜病变的流行病研究)[J]. Chin J Ocul Fundus Dis (中华眼底病杂志). 2010;26(2):109-12.
**DR_C14**	Xiang-wen Shu, Yu Wang, Chuan-feng Fan, et al. 舒相汶, 王玉, 范传峰, 盛艳娟, 张华, 吴昌龙. Epidemiology study on the prevalence rate and risk factors of diabetic retinopathy in rural residents in Shandong province (山东省农村人群糖尿病视网膜病变的流行病学调查)[J]. Chin J Ocul Fundus Dis (中华眼底病杂志). 2010;26(2):113-5.
**DR_C15**	Li-tao Gao. 高丽涛. Analysising the prevalence and risk factors of diabetic retinopathy in Shenyang Heping district (沈阳和平区糖尿病视网膜病变的患病率及相关危险因素分析)[D]. China Medical University (中国医科大学). 2010.
**DR_C16**	Bing-zhen Li, Yu-ling Liu, Liang Han, et al. 李炳震, 刘瑜玲, 韩亮, et al. Epidemiological survey of diabetic retinopathy in Shunyi district of Beijing (北京市顺义 40 岁及以上人群糖尿病视网膜病变的流行病学调查)[J]. Chin J Exp Ophthalmol (中华实验眼科杂志). 2011;29(8):747-52.
**DR_C17**	Yue-dong Hu. 胡悦东. Prevalence of diabetic retinopathy in Liaoning province China and the expression and mechanism of MicroRNA in patients with proliferative diabetic retinopathy (辽宁省自然人群糖尿病视网膜病变现况研究及增殖性糖尿病视网膜病变患者 miRNA 表达及其机制的研究)[D]. China Medical University (中国医科大学). 2011.
**DR_C18**	Qing-xia Liu, Pei-feng Liang, Lai-jun Xu, et al. 刘青霞, 梁沛枫, 胥来军, et al. Epidemiology research of diabetic retinopathy in Ningxia region (宁夏地区糖尿病视网膜病变的流行病学研究)[J]. Int Eye Sci (国际眼科杂志). 2012(08):1566-9.
**DR_C19**	Jun Zhang. 张俊. Epidemiological study on diabetic retinopathy and the analysis of correlative factors in Luzhou city, Sichuan Province (四川省泸州市糖尿病视网膜病变的流行病学调查及相关因素分析)[D]. Southwest Medical University (泸州医学院 西南医科大学). 2012.
**DR_C20**	Ming-xia Yuan, Zhong Xin, Jian-ping Feng, et al. 袁明霞, 信中, 冯建萍, et al. A population-based prevalence survey and risk factor analysis of diabetic retinopathy in Beijing Changping District (北京市昌平区自然人群糖尿病视网膜病变患病率调查及危险因素分析)[J]. Journal of Capital Medical University (首都医科大学学报). 2012(05):669-75.
**DR_C21**	Ying Cui. 崔颖. Epidemiological study of diabetic retinopathy in Dongguan city, Guangdong province (广东省东莞市糖尿病视网膜病变流行病学研究)[D].Southern Medical University (南方医科大学). 2013.
**DR_C22**	Na Li, Xiu-fen Yang, Yu Deng, et al. 李娜, 杨秀芬, 邓禹, et al. Diabetes self-management and its association with diabetic retinopathy in patients with type 2 diabetes (2 型糖尿病患者自我管理水平与糖尿病视网膜病变的相关性研究)[J]. Chin J Ophthalmol (中华眼科杂志). 2013;49(6):500-6.
**DR_C23**	Tian-hua Xie, Jing, Zhu, Dong-hong Fu, et al. 谢田华, 朱靖, 傅东红, et al. Prevalence of diabetic retinopathy in residents aged 50 and above in Binhu community of Wuxi city (无锡市滨湖区 50 岁及以上人群糖尿病视网膜病变患病情况调查)[J]. Chin J Ocul Fundus Dis (中华眼底病杂志). 2013;29(5):495-8.

**DR_C24**	Peng-cheng Wu, Wen-fang Zhang, Peng Lv, et al. 吴鹏程, 张文芳, 律鹏, 陈盛举, 陶明. Epidemical survey of relative factors of retinal vessels disease of the native Tibetan among the people aged 40 and above in Maqin county, Qinghai province (青海省玛沁县 40 岁以上世居藏族人群视网膜血管性疾病相关因素的流行病学调查)[J]. Int Eye Sci (国际眼科杂志). 2014(07):1288-91.
**DR_C25**	Xiao-yan Mou, Ying Wang, Lin Fu, et al. 牟晓燕, 王颖, 付琳, 王楠, 杨晶, 马莉. Prevalence and risk factors of diabetic retinopathy in type 2 diabetic patients in an island population in Dalian (海岛人群糖尿病患者视网膜病变流行现状及影响因素分析)[J]. Modern Preventive Medicine (现代预防医学). 2014(20):3655-8.
**DR_C26**	Lei Shao, Ya-xing Wang, Jie Chen, et al. 邵蕾, 王亚星, 徐捷, et al. Subfoveal choroidal thickness of Chinese aged over 50 years and patients with diabetes mellitus and glaucoma (北京地区 50 岁以上人群及糖尿病和青光眼患者的脉络膜厚度及其影响因素)[J]. Chin J Ophthalmol (中华眼科杂志). 2014(6):414-20.
**DR_C27**	Xue-feng Jiang, Zheng-yu Zhu, Yong-wu He, et al. 江雪丰, 褚征宇, 何勇武, 程玲, 张宏娣. Prevalence and risk factors of Nanchang community diabetic retinopathy (南昌市社区糖尿病视网膜病变患病率及危险因素分析)[J]. Rec Adv Ophthalmol (眼科新进展). 2015(11):1047-50.
**DR_C28**	Wei-chao Wang, Jie Zhang, Hong Wang, et al. 王伟超, 张洁, 王虹, et al. Relationship between the levels of tear fluid TNF-α, serum TNF-α and serum HbA1c and diabetic retinopathy in middle-aged and elderly diabetes patients (社区中老年糖尿病患者泪液和血清肿瘤坏死因子 α 及血清糖化血红蛋白与糖尿病视网膜病变的关系)[J]. Chinese General Practice (中国全科医学). 2015(35):4288-92.
**DR_C29**	Bing-lin Ye, Min Lu, Hao-ying Tang, et al. 叶炳林, 卢敏, 唐浩英, et al. An epidemiological investigation on diabetic retinopathy in people aged 60 years and above in Sanshui district, Foshan city* (佛山市三水区 60 岁及以上人群糖尿病视网膜病变的流行病学调查)[J]. Heilongjiang Medical Journal (黑龙江医学). 2016(04):336-8.
**DR_C30**	Bo-jie Hu, Qiu-hai Lu, Jian-qun Zhan. 胡博杰, 路秋海, 谌建群. Epidemiological survey on diabetic retinopathy in Uyghur patients aged 40 or older with type 2 diabetes mellitus in Hotan region of Xinjiang (新疆和田地区 40 岁及以上维吾尔族 2 型糖尿病患者糖尿病视网膜病变流行病学调查)[J]. Journal of Logistics University of PAP (Medical Sciences) (武警后勤学院学报(医学版)). 2016(08):664-6.
**DR_C31**	Gui-sen Zhang, Jilitu Morige, Feng-mei Ren, et al. 张贵森, 莫日格吉力吐, 任凤梅, 惠延年. Epidemiological investigation of diabetic retinopathy in Hohhot (呼和浩特地区糖尿病视网膜病变流行病学调查)[J]. Chin J Pract Ophthalmol (中国实用眼科杂志). 2017;35(4):428-33.
**DR_C32**	Xie XW, Xu L, Wang YX, Jonas JB. Prevalence and associated factors of diabetic retinopathy. The Beijing Eye Study 2006. Graefes Arch Clin Exp Ophthalmol 2008;246(11):1519-26.
**DR_C33**	Xie XW, Xu L, Jonas JB, Wang YX. Prevalence of diabetic retinopathy among subjects with known diabetes in China: the Beijing Eye Study. EUR J OPHTHALMOL 2009;19(1):91-9.
**DR_C34**	Wang FH, Liang YB, Peng XY, et al. Risk factors for diabetic retinopathy in a rural Chinese population with type 2 diabetes: the Handan Eye Study. ACTA OPHTHALMOL 2011;89(4):e336-43.
**DR_C35**	Wang B, Liu M, Li X, et al. Cutoff Point of HbA1c for Diagnosis of Diabetes Mellitus in Chinese Individuals. PLOS ONE 2016;11(11):e166597.
PHCM-based studies	

**DR_P1**	Li-ping Liu, Ji-wei Zhu, Yi Xiong, et al. 刘丽萍, 朱吉伟, 熊毅, 陈雁, 黄红儿. The prevalence and related factors of diabetic retinopathy in Shanghai Songnan community (上海市淞南社区糖尿病居民中糖尿病视网膜病变患病率及其影响因素调查分析)[J]. Chin J Ocul Dundus Dis (中华眼底病杂志). 2015;31(2):126-9.
**DR_P2**	Chen Liang, Rong Shi, Jing-fen Zhu, et al. 梁辰, 施榕, 朱静芬, et al. Prevalence of diabetic retinopathy of type 2 diabetes mellitus patients in Shanghai Pudong New Area and influencing factors (上海市浦东新区社区 2 型糖尿病患者糖尿病性视网膜病变的患病情况及影响因素调查)[J]. Chinese General Practice (中国全科医学). 2016(04):474-8.
**DR_P3**	Xiao-hua Zhang, Cheng-jun Liu, Wei Zhou, et al. 章小花, 刘成军, 周伟, et al. Survey and analysis on diabetic retinopathy and its factors among type 2 diabetic patients in the Southern Suburb of Pudong New Area (浦东新区南郊 2 型糖尿病患者视网膜病变及相关因素调查分析)[J]. Chinese Primary Health Care (中国初级卫生保健). 2016(04):52-4.
**DR_P4**	Xu J, Wei WB, Yuan MX, et al. Prevalence and risk factors for diabetic retinopathy: the Beijing Communities Diabetes Study 6. Retina 2012;32(2):322-9.
**DR_P5**	Pan CW, Wang S, Qian DJ, Xu C, Song E. Prevalence, Awareness, and Risk Factors of Diabetic Retinopathy among Adults with Known Type 2 Diabetes Mellitus in an Urban Community in China. Ophthalmic Epidemiol 2017;24(3):188-94.
Registry-based study	
**DR_R1**	Wei-jie Wang, Yi-nan Liu, Yu-jie Yan, et al. 王伟杰, 刘奕男, 严玉洁, 姚保栋, 周凡, 郦伦强. The investigation of risk factors of diabetic retinopathy in type 2 diabetes mellitus in Minhang, Shanghai (上海市闵行区 2 型糖尿病患者糖尿病视网膜病变患病率及相关危险因素分析)[J]. Chinese Primary Health Care (中国初级卫生保健). 2015(08):66-8.

Note: The Chinese publication list employed the journals' official English names or abbreviations, English titles were obtained from journals or literature databases (CNKI, Wanfang and CBM); Where official English translation of journal names is not available, a pinyin title is adopted; where the English translation of titles is not available, I translated the titles, labelled with "" and marked as green; PHCM, Primary Health Care Management.*

Table S4. Detailed characteristics of the included studies on the prevalence of and risk factors for DR in China (n=41)

Study ID	Study	Province	Region	Study Year	Gender	Urban/Rural	Grading system of DR	For Prevalence analysis	For risk factor analysis	General sample size	Diabetic sample size	Any DR	NPDR	PDR
Community-based studies														
**DR_C1**	Zhang XM et al. (1996)	Guangxi	South Central China	1994	Both	Mixed	NCOFD	Yes	No	11866	275	27	26	1
**DR_C2**	He SZ et al. (1997)	Beijing	North China	1994*	Both	Urban	NOFDG	Yes	Yes	29938	534	90	88	2
**DR_C3**	Li SL et al. (1998)	Anhui	East China	1994	Mixed	Mixed	NCOFD	Yes	No	11618	216	67	-	-
**DR_C4**	Li N et al. (1999)	Xinjiang	Northwest China	1995	Mixed	Urban	CMB	Yes	No	-	276	54	52	2
**DR_C5**	Wang GL et al. (2001)	Beijing	North China	1994	Mixed	Mixed	NCOFD	Yes	No	20682	293	33	28	5
**DR_C6**	Yang X et al. (2004)	Xinjiang	Northwest China	2000	Mixed	Urban	NCOFD	Yes	No	6542	224	26	-	-
**DR_C7**	Liu JP (2006)	Tianjin	North China	2005	Both	Mixed	ICDRDSS	Yes	No	23212	650	106	102	4
**DR_C8**	Hu HY et al. (2007)	Shanghai	East China	2006	Both	Urban	ICDRDSS	No	Yes	-	672	154	145	9
**DR_C9**	Dong B et al. (2008)	Guangdong	South Central China	2005*	Mixed	Urban	NCOFD	Yes	No	5731	196	22	-	-
**DR_C10**	Liu L (2009)	Liaoning	Northeast China	2007	Both	Urban	NCOFD	Yes	Yes	1534	137	17	17	0
**DR_C11**	Zhang HX et al. (2009)	Shanghai	East China	2005	Both	Urban	ICDRDSS	Yes	Yes	-	642	78	76	2
**DR_C12**	Xin Z et al. (2010)	Beijing	North China	2008	Mixed	Rural	ICDRDSS	Yes	Yes	1293	114	27	25	2
**DR_C13**	Wang HB et al. (2010)	Shanxi	North China	2008	Mixed	Rural	NOFDG	Yes	No	57500	2632	986	-	-
**DR_C14**	Shu XW et al. (2010)	Shandong	East China	2007	Both	Rural	ICDRDSS	Yes	No	16330	689	181	136	45
**DR_C15**	Gao LT (2011)	Liaoning	Northeast China	2009	Both	Urban	NCOFD	Yes	No	740	66	6	-	-
**DR_C16**	Li BZ et al. (2011)	Beijing	North China	2007	Both	Mixed	ICDRDSS	Yes	No	4167	445	130	124	6
**DR_C17**	Hu YD (2011)	Liaoning	Northeast China	2007	Both	Mixed	ETDRS	Yes	Yes	3201	339	57	-	-
**DR_C18**	Liu QX et al. (2012)	Ningxia	Northwest China	2009*	Mixed	Mixed	ICDRDSS	Yes	No	3001	76	13	-	-
**DR_C19**	Zhang J (2012)	Sichuan	Southwest China	2011	Both	Urban	NCOFD	Yes	Yes	7478	1374	214	194	20
**DR_C20**	Yuan MX et al. (2012)	Beijing	North China	2010	Mixed	Mixed	ICDRDSS	Yes	No	8155	614	61	59	2
**DR_C21**	Cui Y (2013)	Guangdong	South Central China	2012	Both	Rural	ICDRDSS	Yes	Yes	8592	1310	235	-	-
**DR_C22**	Li N et al. (2013)	Beijing	North China	2010	Both	Urban	ETDRS	No	Yes	-	1100	353	300	53
**DR_C23**	Xie TH et al. (2013)	Jiangsu	East China	2010	Both	Urban	ICDRDSS	Yes	No	6150	663	36	34	2
**DR_C24**	Wu PC et al. (2014)	Qinghai	Northwest China	2012	Mixed	Rural	NCOFD	Yes	No	2511	12	5	-	-

**DR_C25**	Mou XY et al. (2014)	Liaoning	Northeast China	2011*	Both	Rural	ICDRDSS	Yes	Yes	-	603	219	217	2
**DR_C26**	Shao L et al. (2014)	Beijing	North China	2011	Mixed	Mixed	ETDRS	Yes	No	3468	246	23	-	-
**DR_C27**	Jiang XF et al. (2015)	Jiangxi	East China	2012	Both	Urban	ICDRDSS	Yes	Yes	9776	730	223	-	-
**DR_C28**	Wang WC et al. (2015)	Hebei	North China	2011	Mixed	Urban	ICDRDSS	No	Yes	1447	396	212	149	63
**DR_C29**	Ye BL et al. (2016)	Guangdong	South Central China	2011	Both	Urban	ETDRS	Yes	Yes	3751	286	82	57	25
**DR_C30**	Hu BJ et al. (2016)	Xinjiang	Northwest China	2014	Mixed	Mixed	ETDRS	Yes	No	7462	618	77	76	1
**DR_C31**	Zhang GS et al. (2017)	Inner Mongolia	North China	2014	Both	Mixed	NOFDG	Yes	No	3967	352	26	25	1
**DR_C32**	Xie XW et al. (2008)	Beijing	North China	2006	Both	Mixed	ETDRS	Yes	Yes	3251	362	101	-	-
**DR_C33**	Xie XW et al. (2009)	Beijing	North China	2001	Mixed	Mixed	ETDRS	No	Yes	4127	232	86	-	-
**DR_C34**	Wang FH et al. (2011)	Hebei	North China	2007	Both	Rural	ETDRS	Yes	No	5597	368	165	-	-
**DR_C35**	Wang B et al. (2016)	Liaoning	Northeast China	2011	Mixed	Urban	ICDRDSS	Yes	No	8391	1809	233	-	-
PHCM-based studies														
**DR_P1**	Liu LP et al. (2015)	Shanghai	East China	2013	Both	Urban	ICDRDSS	No	Yes	-	1120	264	261	3
**DR_P2**	Liang C et al. (2016)	Shanghai	East China	2014	Both	Urban	ICDRDSS	No	Yes	-	2083	445	-	-
**DR_P3**	Zhang XH et al. (2016)	Shanghai	East China	2013*	Both	Urban	ICDRDSS	No	Yes	-	1437	151	147	4
**DR_P4**	Xu J et al. (2012)	Beijing	North China	2009	Both	Urban	ETDRS	No	Yes	-	2007	496	429	67
**DR_P5**	Pan CW et al. (2017)	Jiangsu	East China	2015	Both	Urban	ETDRS	No	Yes	-	880	158	-	-
Registry-based study														
**DR_R1**	Wang WJ et al. (2015)	Shanghai	East China	2012*	Both	Urban	ICDRDSS	No	Yes	-	2152	736	687	49

Note: "-" represents unavailable data; "" indicates studies whose survey year was imputed. PHCM, Primary Health Care Management; NCOFD, National Conference on Ocular Fundus Diseases; NOFDG, National Ocular Fundus Diseases Group; CBM, China Medical Board; ICDRDSS, International Clinical Diabetic Retinopathy Disease Severity Scale; ETDRS, Early Treatment of Diabetic Retinopathy Study.*

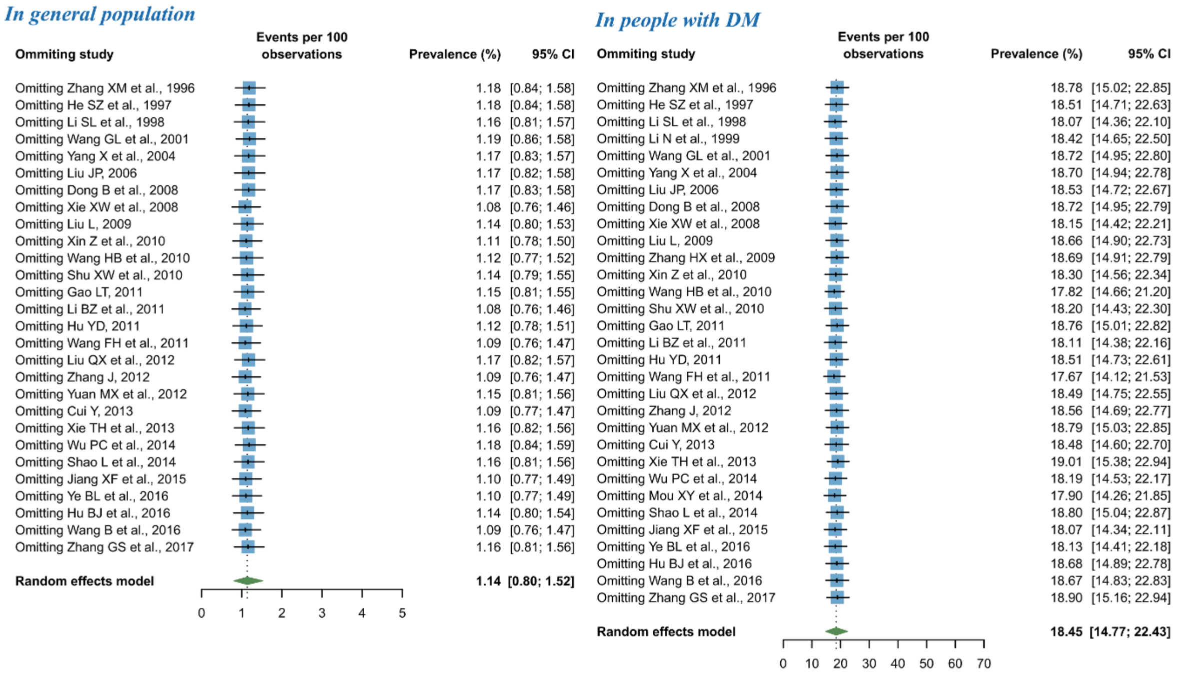

Figure S3. Leave-one-out sensitivity analysis of the influence of single study on the pooled prevalence of any DR in general population and people with DM

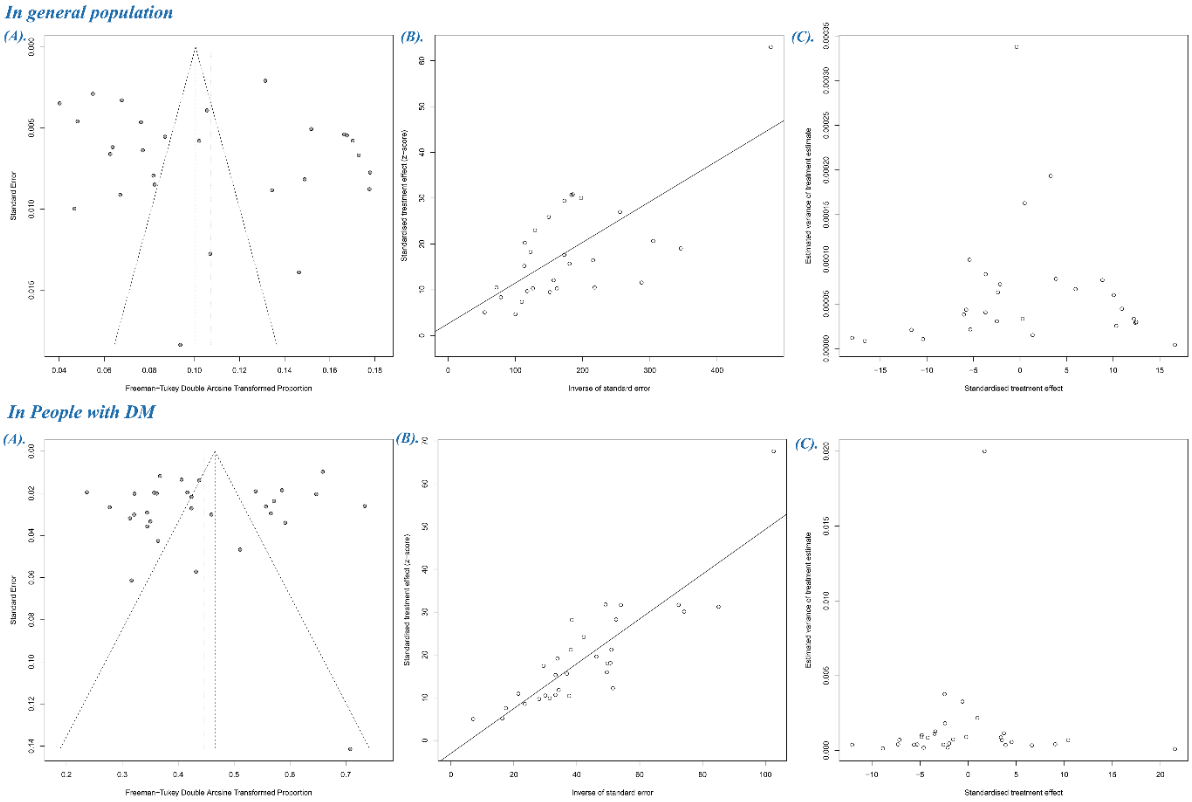

Figure S4. Publication bias of the studies on the prevalence of any DR in general population and people with DM

Note: (A) Funnel plot; (B) Egger's test (studies on DR prevalence in general population: t= 0.703, p= 0.488; studies on DR prevalence in people with DM: t= -1.128, p= 0.269); (C) Begg's test (*studies on DR prevalence in general population: z= 0.553, p= 0.580; studies on DR prevalence in people with DM: z=- 0.493, p= 0.622).*

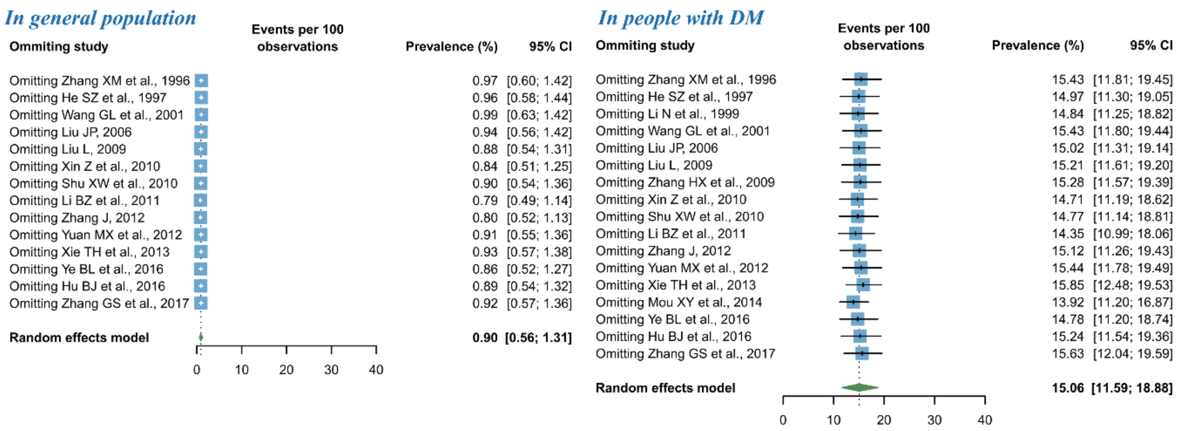

Figure S5. Leave-one-out sensitivity analysis of the influence of single study on the pooled prevalence of NPDR in general population and people with DM

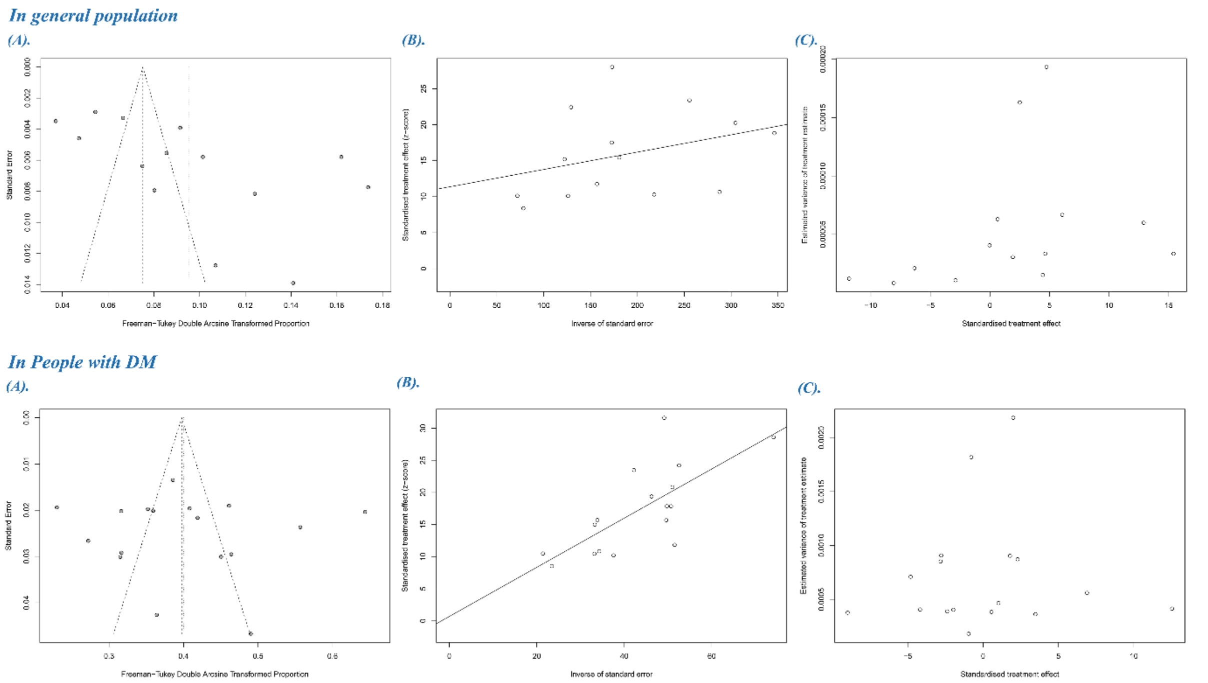

Figure S6. Publication bias of the studies on the prevalence of NPDR in general population and people with DM

Note: (A) Funnel plot; (B) Egger's test (studies on DR prevalence in general population: t= 2.858, p= 0.014; studies on DR prevalence in people with DM: t= 0.162, p= 0.873); (C) Begg's test (studies on DR prevalence in general population: z= 2.135, p= 0.033; studies on DR prevalence in people with DM: z= 0.494, p= 0.621).

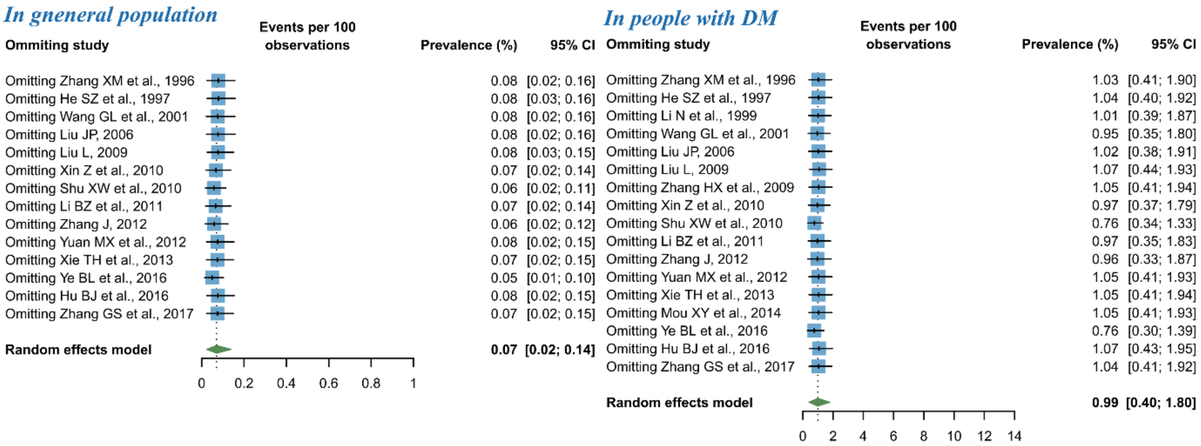

Figure S7. Leave-one-out sensitivity analysis of the influence of single study on the pooled prevalence of PDR in general population and people with DM

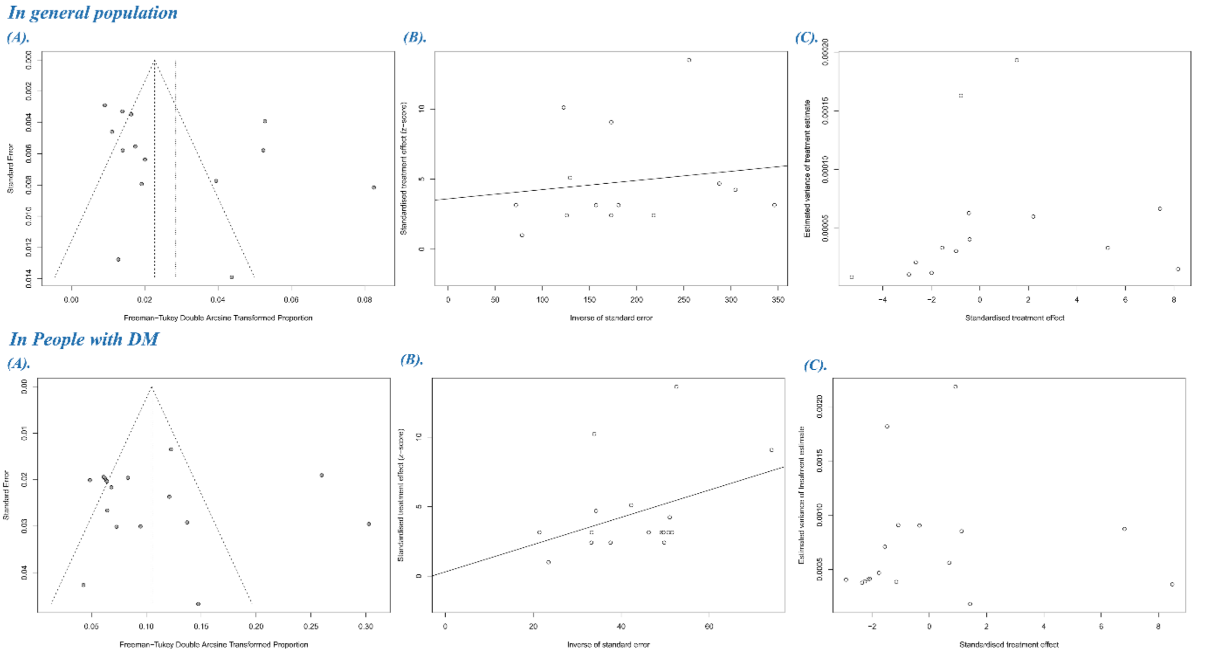

Figure S8. Publication bias of the studies on the prevalence of PDR in general population and people with DM

Note: (A) Funnel plot; (B) Egger's test (studies on DR prevalence in general population: t= 1.4635, p= 0.169; studies on DR prevalence in people with DM: t= 0.11095, p= 0.9131); (C) Begg's test (studies on DR prevalence in general population z= 2.1351, p= 0.03276; studies on DR prevalence in people with DM: z= 1.2358, p= 0.2165).

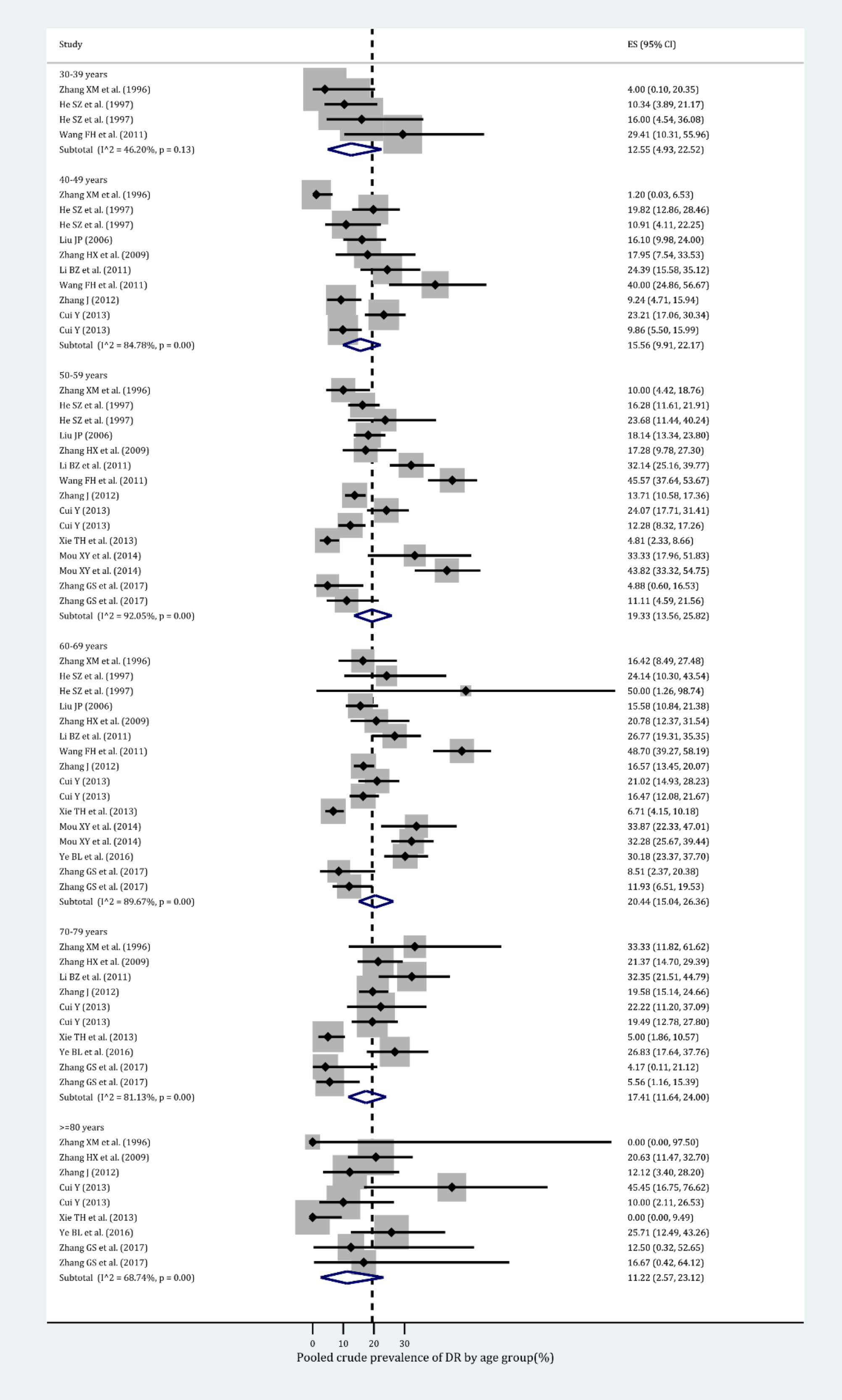

Figure S9. The prevalence of any DR in people with DM in different age groups, by random-effects meta-analysis

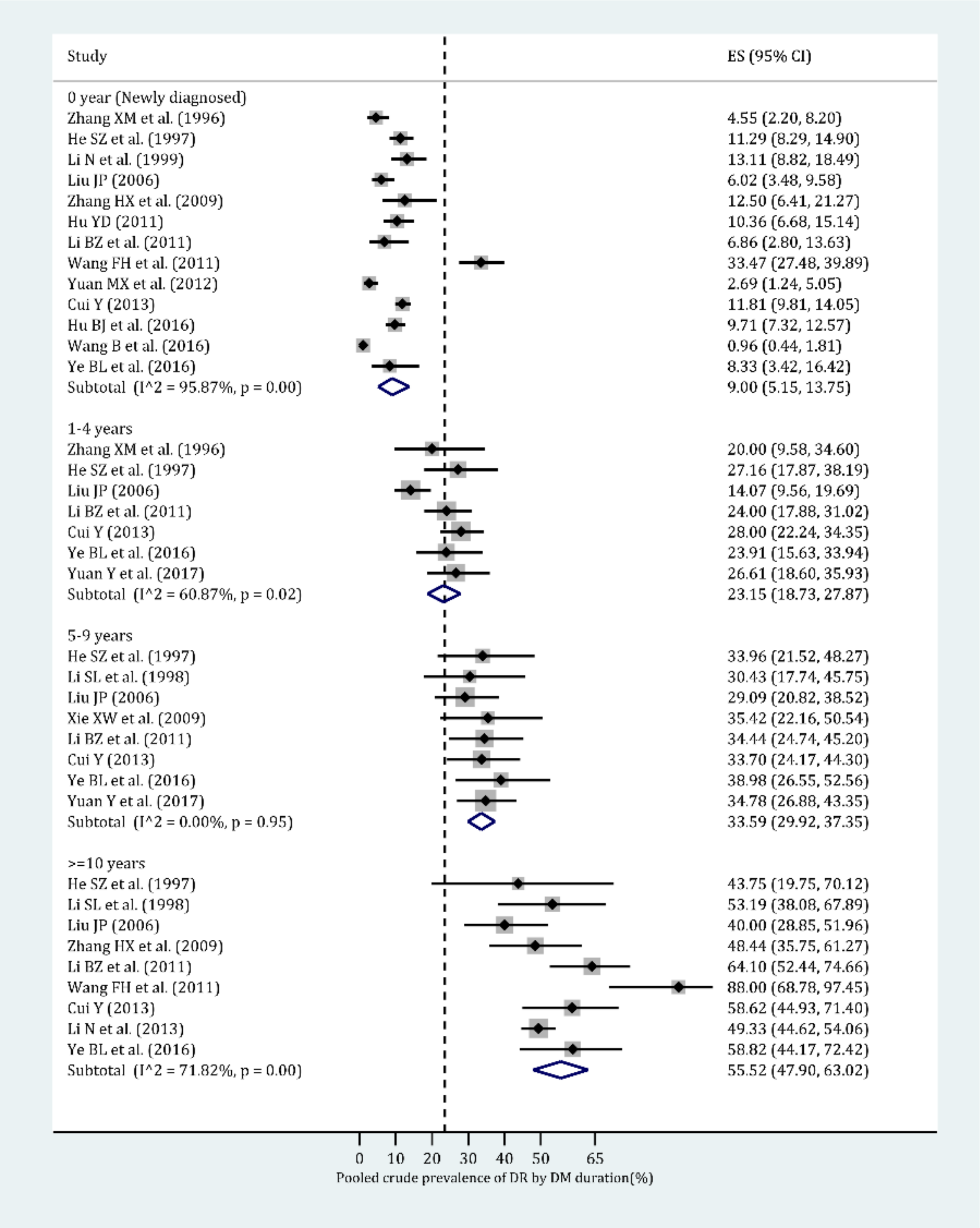

Figure S10. The prevalence of any DR in people with DM in different DM duration groups, by random-effects meta-analysis

Table S5. Full list of studies reporting risk factors for DR in China (n=21)

Study ID	Study	Reported risk factor
DR_P2	He SZ et al. (1997)	DM_Duration (per year), Dietary control, FBG (mmol/l), HbA1c (%), Scr, Ucr, TC (mmol/l), TG (mmol/l)
DR_P8	Hu HY et al. (2007)	DM_Duration (per year), FBG (mmol/l)
DR_P10	Liu L (2009)	DM_Duration group comparison (>10 vs. <5), FBG (mmol/l), BMI (kg/m^2), Drinking
DR_P12	Xin Z et al. (2010)	DM_Duration (per year), FBG (mmol/l), SBP (mmHg)
DR_P19	Li BZ et al. (2011)	DM_Duration (per year), FBG (mmol/l), BMI (kg/m^2), Education (literacy vs. illiteracy), Insulin treatment
DR_P21	Hu YD (2011)	DM history, Income
DR_P23	Zhang J (2012)	DM_Duration (per year), HbA1c (%), HDL (mmol/l), Hypertension
DR_P28	Cui Y (2013)	DM_Duration group comparison (>=10 vs. 0), FBG level group comparison (>=7.0 vs. <5.6), HbA1c level group comparison (>=6.5 vs. <6.5), SBP level group comparison (>=140 vs. <120), Male gender
DR_P29	Li N et al. (2013)	DM_Duration group comparison (>=10 vs. <10), HbA1c level group comparison (>=7.0 vs. <7.0), Low-education, Insulin treatment, Low income, Male gender
DR_P34	Mou XY et al. (2014)	DM_Duration (per year), FBG (mmol/l), BMI (kg/m^2), Insulin treatment, Weight (kg), Hyperlipidemia
DR_P36	Jiang XF et al. (2015)	Age (per year), DM_Duration group comparison (>10 vs. <=5), 2h-PBG, TC (mmol/l), TG (mmol/l), BMI (kg/m^2), SBP (mmHg), DBP (mmHg), Male gender, 24h UP (mg.d)
DR_P37	Wang WC et al. (2015)	Age group comparison (>65 vs. 45-65), FBG (mmol/l), 2h-PBG, HbA1c (%), TG (mmol/l), BMI (kg/m^2), WHR, DM family history, Male gender, Tear TFN-α (ng/l), Blood TFN-α (ng/l)
DR_P38	Ye BL et al. (2016)	DM_Duration group comparison (per year increase vs. 0), FBG (mmol/l), Education (literacy vs. illiteracy), Insulin treatment
DR_P43	Xie XW et al. (2008)	DM_Duration (per year), FBG (mmol/l), TC (mmol/l), SBP (mmHg), Insulin treatment vs. diet only, HDL (mmol/l), Rural, Hyperopic refractive error (per diopter)
DR_P44	Xie XW et al. (2009)	DM_Duration group comparison (>=20 vs. 5), Insulin treatment vs. diet only, Rural
DR_M7	Liu LP et al. (2015)	Age (per year), DM_Duration (per year), SBP (mmHg), Insulin treatment
DR_M9	Liang C et al. (2016)	Age of DM onset, DM_Duration (per year), 2h-PBG, HbA1c (%)
DR_M10	Zhang XH et al. (2016)	DM_Duration group comparison (>15 vs. <5), FBG level group comparison (>6.8 vs. <6.8)
DR_M13	Xu J et al. (2012)	Age (per year), DM_Duration (per year), HbA1c (%), BMI (kg/m^2), SBP (mmHg), BUN (mmol/l)
DR_M15	Pan CW et al. (2017)	Age (per year), DM_Duration (per year), HbA1c (%), Smoking, DM family history, Hypertension, Male gender
DR_R1	Wang WJ et al. (2015)	DM_Duration (per year), HbA1c (%)

Note: DM, diabetes mellitus; FBG, fasting blood glucose; PBG, postprandial blood glucose; HbA1c, glycated haemoglobin A1c; Scr, serum creatinine; Ucr, urine creatinine; TC, total cholesterol; TG, triglyceride; BMI, body mass index; SBP, systolic blood pressure; HDL, high-density lipoprotein; DBP, diastolic blood pressure; UP, urine protein; WHR, waist-to-hip ratio; TFN-α, Tumor necrosis factor alpha; BUN, blood urea nitrogen. Some studies didn't specify the units for some specific risk factors, in circumstances where assumptions could not be made, risk factors without units were not included for subsequent meta-analyses.

Table S6. Meta analyses of 11 risk factors for any DR in people with DM

Risk factor 1-Advanced age (per year increase)

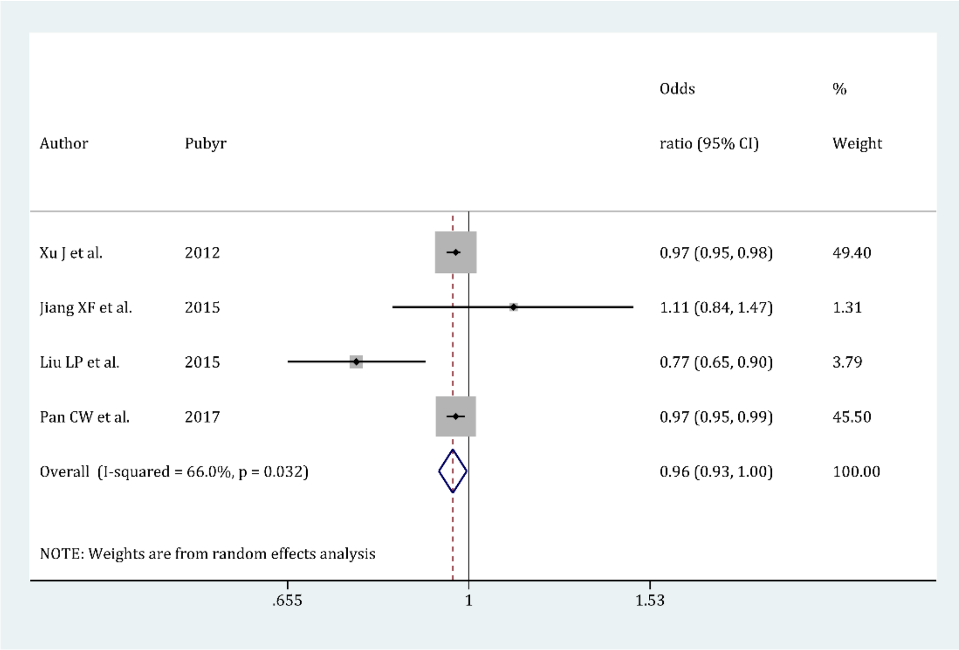

Risk factor 2-Male

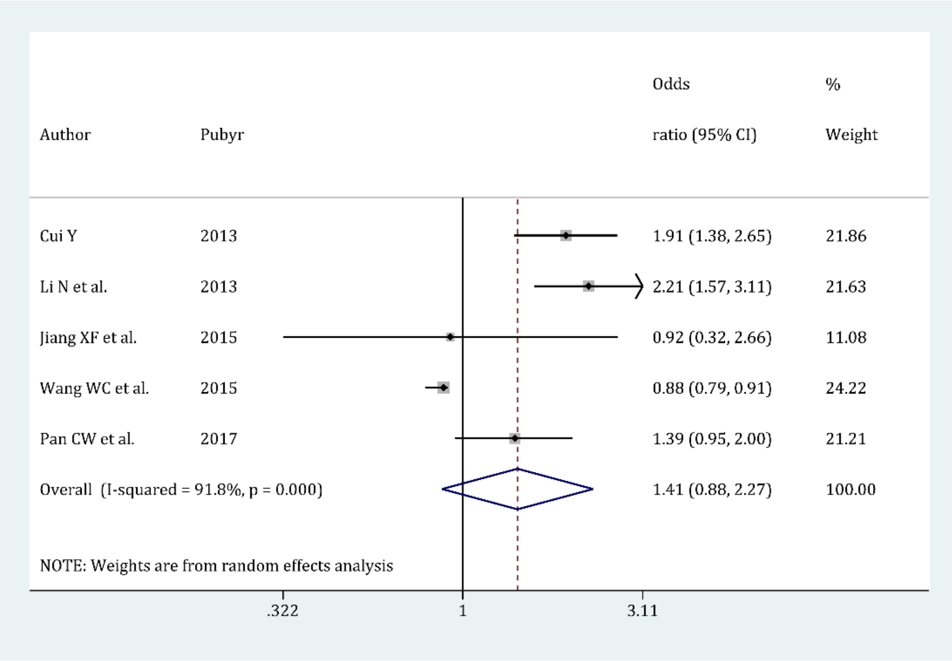

Risk factor 3-DM duration (per year increase)

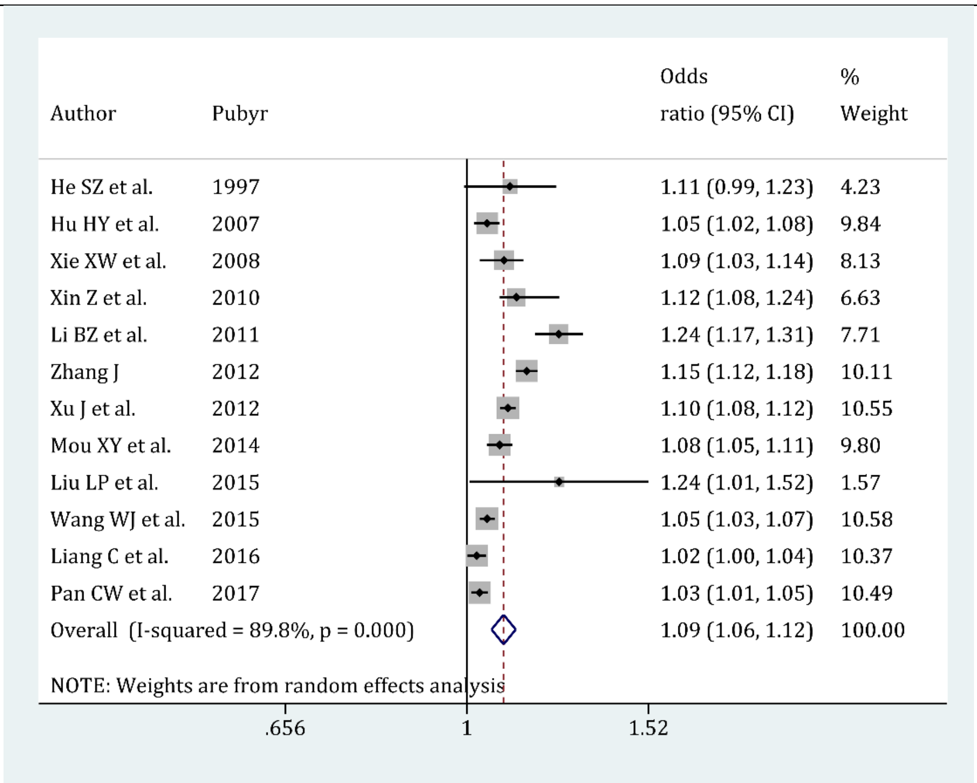

Risk factor 4-Insulin treatment

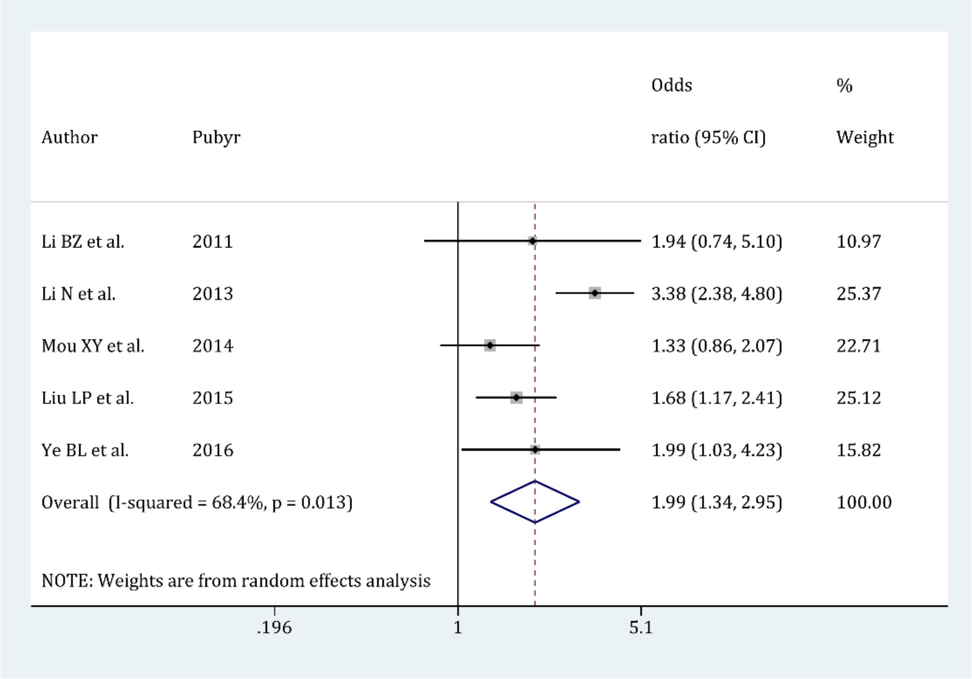

Risk factor 5-FBG (per mmol/l increase)

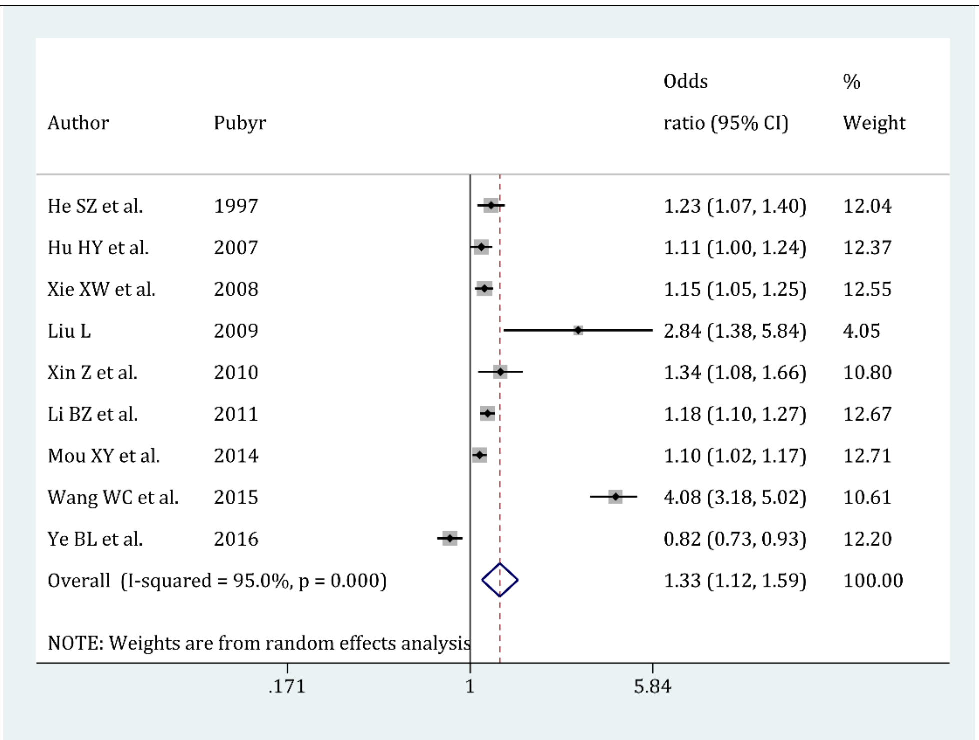

Risk factor 6-2h PBG (per mmol/l increase)

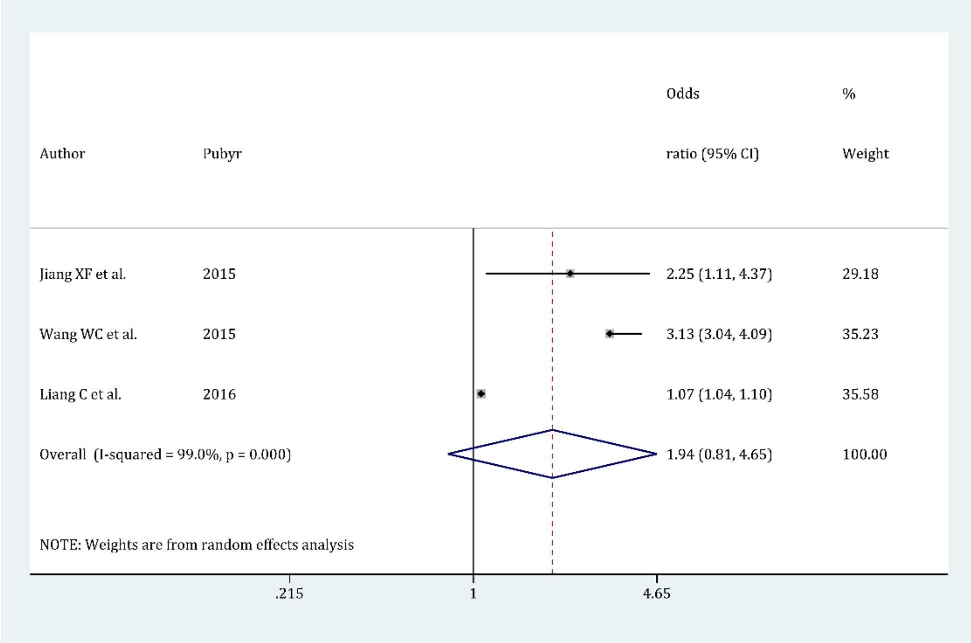

Risk factor 7-HbA1c (per % increase)

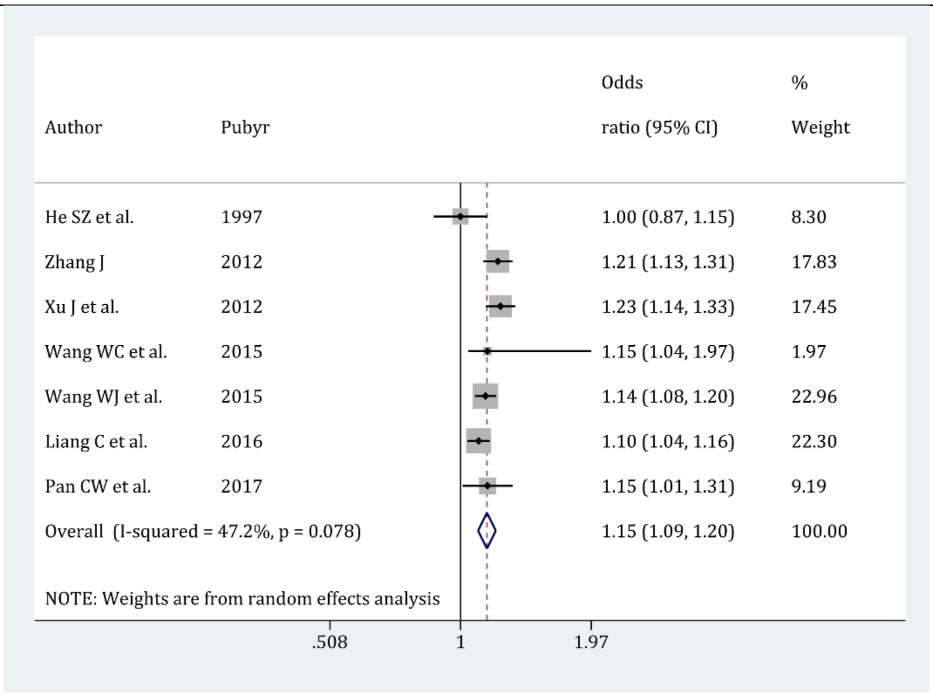

Risk factor 8-TC (per mmol/l increase)

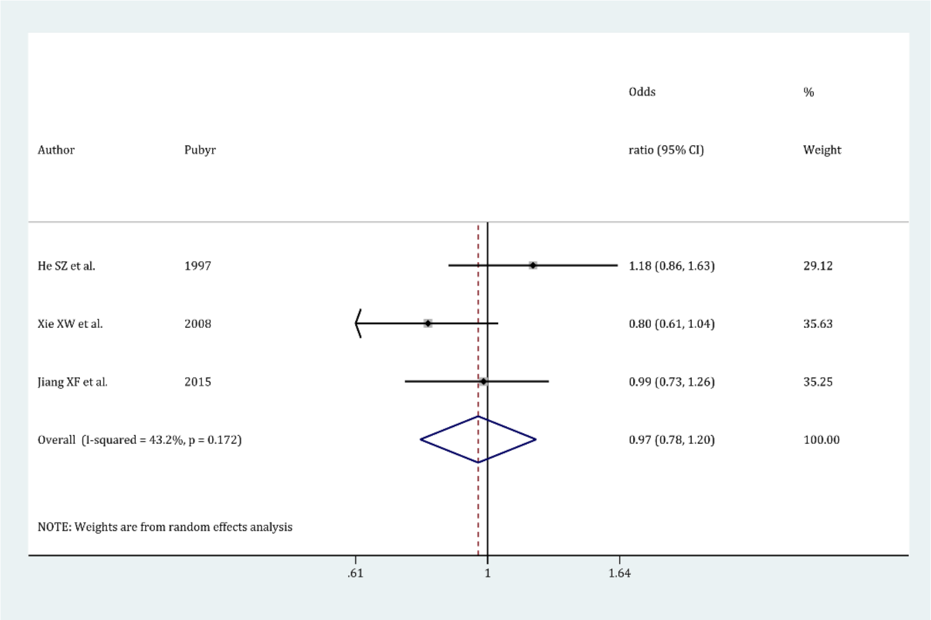

Risk factor 9-TG (per mmol/l increase)

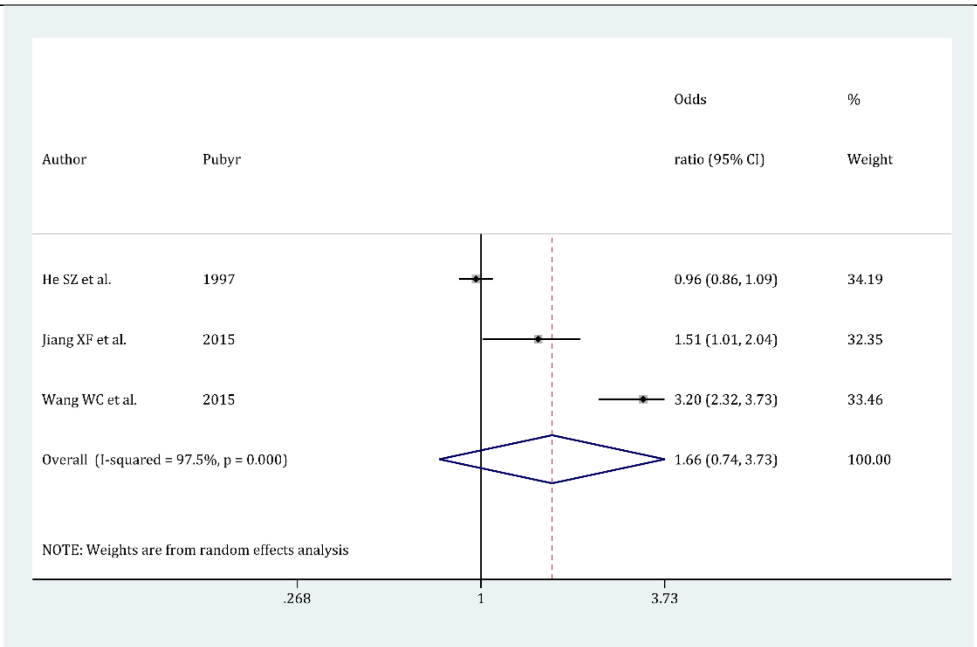

Risk factor 10-BMI (per kg/m2 increase)

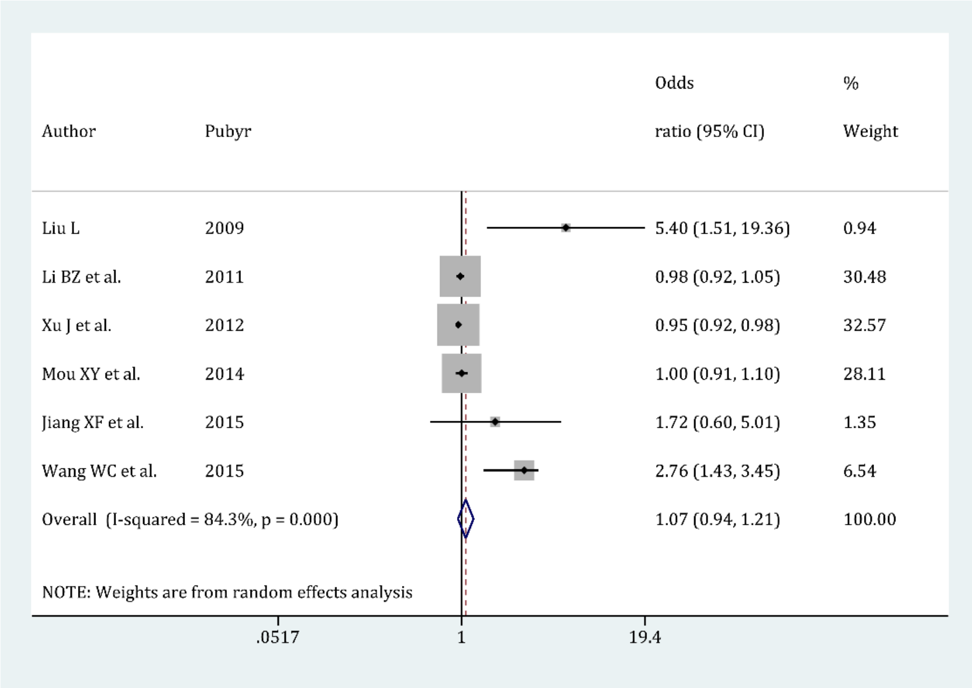

Risk factor 11-SBP (per mmHg increase)

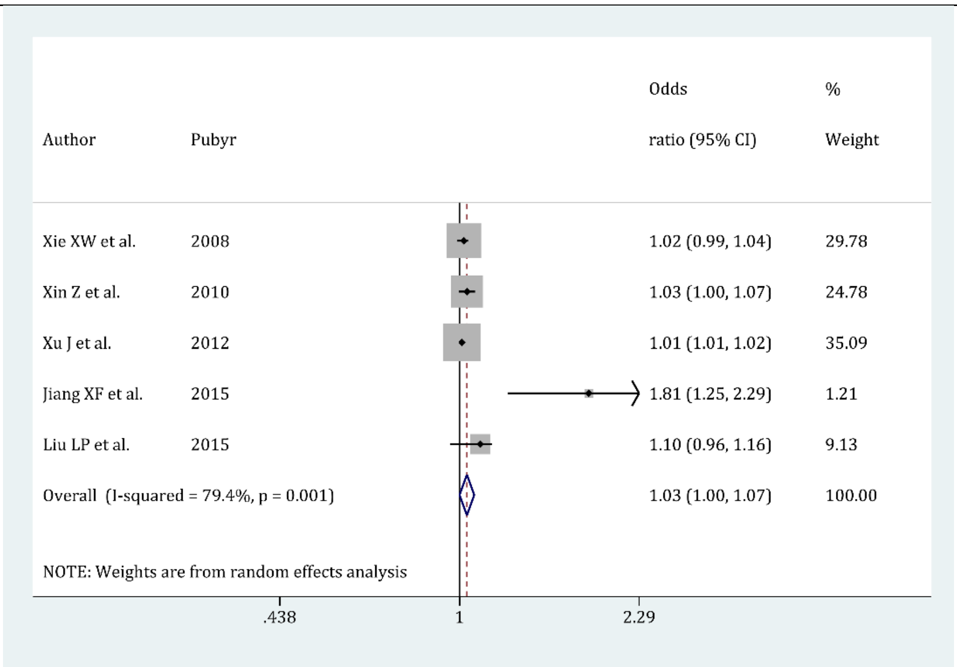
Odds
%
Author
Pubyr
ratio (95% CI)
Weight
Xie XW et al.
2008
1.02 (0.99, 1.04)
29.78
Xin Z et al.
2010
1.03 (1.00, 1.07)
24.78
Xu J et al.
2012
1.01 (1.01, 1.02)
35.09
Jiang XF et al.
2015
1.81 (1.25, 2.29)
1.21
Liu LP et al.
2015
1.10 (0.96, 1.16)
9.13
Overall (I-squared = 79.4%, p = 0.001)
1.03 (1.00, 1.07)
100.00
NOTE: Weights are from random effects analysis
.438
1
2.29

Table S7. Mean fasting blood glucose, mean haemoglobin A1c and prevalence of diabetes mellitus in middle-aged and older Chinese, CHARLS 2011

Characteristic	Study population (11308)	Mean FBG, mmol/l (95% CI)	Mean HbA1c, % (95% CI)	Prevalence of DM, % (95% CI)	Proportion within DM, % (95% CI)	
					KDM	NDM
Age group						
45-49 years	2109 (18.65)	5.85±1.62	5.18±0.77	10.76 (8.59-13.40)	30.63 (23.96-38.21)	69.37 (61.79-76.04)
50-59 years	4007 (35.44)	6.09±1.93	5.26±0.84	16.06 (14.14-18.19)	40.22 (33.53-47.28)	59.78 (52.72-66.47)
60-69 years	3285 (29.05)	6.15±2.11	5.33±0.90	19.99 (17.29-22.99)	50.35 (40.94-59.74)	49.65 (40.26-59.06)
70-79 years	1548 (13.69)	6.16±1.95	5.29±0.82	21.78 (17.91-26.23)	45.48 (31.82-59.86)	54.52 (40.14-68.18)
≥80 years	359 (3.17)	6.25±1.05	5.24±0.59	25.25 (13.64-41.94)	10.17 (3.83-24.35)	89.83 (75.65-96.17)
Gender						
Male	5349 (47.30)	6.08±1.84	5.23±0.73	16.78 (14.54-19.28)	37.32 (32.55-42.35)	62.68 (57.65-67.45)
Female	5959 (52.70)	6.07±1.93	5.30±0.92	17.64 (15.98-19.43)	44.20 (36.57-52.10)	55.80 (47.90-63.43)
Setting						
Urban	4210 (37.23)	6.14±1.65	5.29±0.75	20.51 (18.02-23.26)	47.28 (40.26-54.41)	52.72 (45.59-59.74)
Rural	7098 (62.77)	6.01±2.07	5.24±0.88	14.09 (12.76-15.54)	32.14 (27.30-37.39)	67.86 (62.61-72.70)
Region						
North China	1656 (14.64)	6.16±2.23	5.22±0.97	20.83 (16.25-26.30)	42.55 (30.92-55.08)	57.45 (44.92-69.08)
Northeast China	804 (7.11)	6.21±1.77	5.35±0.80	17.50 (13.27-22.72)	44.75 (35.06-54.86)	55.25 (45.14-64.94)
East China	3420 (30.24)	6.04±1.77	5.20±0.81	15.97 (13.93-18.25)	46.61 (39.41-53.94)	53.39 (46.06-60.59)
South Central China	2570 (22.73)	6.01±1.44	5.29±0.75	18.95 (15.09-23.53)	39.28 (25.76-54.67)	60.72 (45.33-74.24)
Southwest China	1930 (17.07)	6.05±2.50	5.33±0.96	13.99 (10.92-17.76)	30.64 (22.16-40.68)	69.36 (59.32-77.84)
Northwest China	928 (8.21)	6.19±2.26	5.29±0.75	16.39 (12.91-20.58)	35.69 (20.29-54.75)	64.31 (45.25-79.71)
Overall	11308 (100.00)	6.07±1.88	5.26±0.83	17.22 (15.57-19.00)	40.92 (35.51-46.56)	59.08 (53.44-64.49)

Note: Calculations were weighted by taking into account the complex survey design and adopted the blood weight with household and individual response adjustment; data were presented as n (%), proportion (95% CI), means ± SD; KDM, known (diagnosed) diabetes mellitus; NDM, newly detected diabetes mellitus.
